# Spatial multi‐omics identifies a NOTCH3‐mediated capillary–mCAF crosstalk driving immune exclusion in hepatocellular carcinoma

**DOI:** 10.1002/imt2.70117

**Published:** 2026-03-17

**Authors:** Fansen Ji, Haochen Li, Qi Wang, Xiaojuan Wang, Jiawei Zhang, Ying Xiao, Huan Li, Hao Liu, Tanqing Long, Boyang Wu, Hao Chen, Haoming Xia, Xinquan Liu, Chuanrui Xu, Yibo Gao, Bingjun Tang, Juan Liu, Shizhong Yang, Jiahong Dong

**Affiliations:** ^1^ Hepatopancreatobiliary Center, Beijing Tsinghua Changgung Hospital, Key Laboratory of Digital Intelligence Hepatology (Ministry of Education), School of Clinical Medicine, Tsinghua Medicine Tsinghua University Beijing China; ^2^ Tsinghua‐Peking Center for Life Sciences Beijing China; ^3^ School of Medicine, Tsinghua Medicine Tsinghua University Beijing China; ^4^ Health Management Center, Beijing Jishuitan Hospital Capital Medical University Beijing China; ^5^ Department of Pathology, Beijing Tsinghua Changgung Hospital Tsinghua University Beijing China; ^6^ School of Pharmacy, Tongji Medical College Huazhong University of Science and Technology Wuhan Hubei China; ^7^ Department of Thoracic Surgery, National Cancer Center/National Clinical Research Center for Cancer/Cancer Hospital Chinese Academy of Medical Sciences and Peking Union Medical College Beijing China; ^8^ Central Laboratory & Shenzhen Key Laboratory of Epigenetics and Precision Medicine for Cancers, National Cancer Center/National Clinical Research Center for Cancer/Cancer Hospital & Shenzhen Hospital Chinese Academy of Medical Sciences and Peking Union Medical College Shenzhen China

**Keywords:** cancer associated fibroblast, hepatocellular carcinoma, immunotherapy, NOTCH3, spatial omics

## Abstract

Fibrosis induced immune exclusion is a hepatocellular carcinoma (HCC) hallmark, underscoring the key role of cancer‐associated fibroblasts (CAFs) in immune regulation. Through HCC spatial multi‐omics data and integrating pan‐cancer scRNA‐seq profiles of CAFs under immune checkpoint blockade (ICB) treatment, we characterized a potential crosstalk between capillaries and CAFs mediated by the NOTCH signaling pathway. Specifically, endothelial DLL4‐NOTCH3 signaling appears to be associated with matrix‐producing CAFs (mCAFs) polarization, leading to extracellular matrix remodeling and the establishment of immune‐restrictive niches that hinder T cell infiltration. Perturbation of NOTCH signaling attenuated mCAF differentiation and enhanced T cell infiltration in vitro, and was associated with improved ICB response in both spontaneous and orthotopic HCC mouse models. Collectively, our findings suggest that capillary‐mCAFs communication through the NOTCH pathway, particularly *NOTCH3* activation, may contribute to fibrosis‐driven immune exclusion in HCC. Targeting this axis could provide a promising strategy to alleviate stromal barriers and potentiate immunotherapy efficacy.

## Highlights

- A spatial multi‐omics atlas of hepatocellular carcinoma (HCC) uncovers a DLL4–NOTCH3 driven capillary–mCAF axis shaping fibrotic, immune‐excluded tumor cores.
- Pan‐cancer single‐cell and spatial integration identifies mCAF polarization as a conserved stromal driver of immune checkpoint blockade resistance.
- Targeting stromal *NOTCH3* disrupts fibrosis, enhances T cell infiltration, and synergizes with anti‐PD‐1 therapy, highlighting a clinically actionable strategy for HCC.

## Graphical abstract

Spatial multi‐omics defines a DLL4–NOTCH3–driven capillary–mCAF axis orchestrating fibrotic remodeling and immune exclusion in hepatocellular carcinoma tumor cores, conferring immune checkpoint blockade resistance. Targeting stromal NOTCH3 disrupts this pro‐fibrotic niche, enhances T cell infiltration, and synergizes with anti‐PD‐1 therapy, highlighting a translatable strategy to overcome immunotherapy resistance.

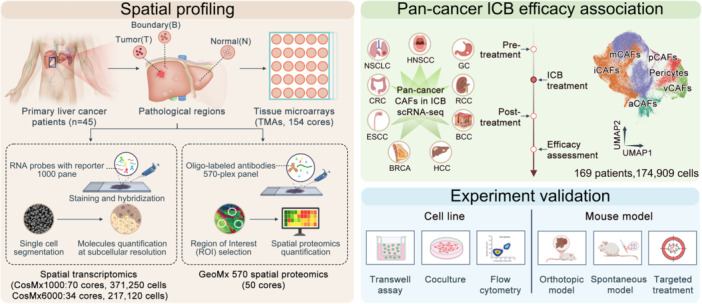

## INTRODUCTION

Hepatocellular Carcinoma (HCC) is the most prevalent form of primary liver cancer and a leading cause of cancer‐related death worldwide [1]. A hallmark pathological feature of HCC is pronounced fibrosis or cirrhosis, often arising from underlying chronic liver diseases such as viral hepatitis, alcohol‐related liver injury, and non‐alcoholic steatohepatitis [2]. The fibrotic background promotes tumor progression and alters the spatial architecture of the tumor microenvironment (TME), impacting disease trajectory and therapeutic response in HCC [3, 4, 5, 6, 7]. In this regard, the fibrotic stroma is increasingly recognized not as a passive scaffold but as a dynamic and formidable barrier that contributes to therapeutic resistance and immune exclusion, posing a major challenge to current HCC management.

Within this fibrotic TME, cancer‐associated fibroblasts (CAFs) are key architects of extracellular matrix remodeling and immune regulation [8, 9]. Previous single‐cell RNA sequencing (scRNA‐seq) studies have revealed remarkable plasticity of CAF subtypes [10, 11, 12, 13, 14, 15], while emerging spatial multi‐omics has further highlighted CAF heterogeneity across multiple cancer types [11, 16, 17, 18, 19]. However, limitations in resolution and multiplexing capacity have hindered the development of a spatially resolved cellular atlas of human HCC [20], impeding detailed investigation of CAF polarization states and their spatially coordinated interactions with immune and vascular components. Moreover, whether functional polarization of distinct CAF subtypes, such as matrix‐producing CAFs (mCAFs) and inflammatory CAFs (iCAFs), establishes stroma‐driven checkpoints that contribute to immune evasion remains insufficiently understood. Given that HCC arises in a fibrosis‐dominated microenvironment, elucidating how vascular cues influence CAF differentiation may provide critical insights into the link between fibrosis, immune exclusion, and therapy resistance, thereby identifying potential strategies for stromal‐targeted intervention.

In this study, we addressed these questions by applying the CosMx™ Spatial Molecular Imager (SMI), a next‐generation spatial transcriptomics platform enabling subcellular‐resolution profiling of thousands of RNA species in both fresh frozen and Formalin‐Fixed, Paraffin‐Embedded (FFPE) samples [21]. Our multi‐dimensional analysis encompassed 154 tissue microarray (TMA) cores comprising 1176 fields of view (FOVs) from 45 patients, systematically mapping the tumor ecosystem across malignant foci, invasive boundaries, and adjacent normal parenchyma. Integrating spatial multi‐omics with public pan‐cancer scRNA‐seq data of CAFs under immune checkpoint blockade (ICB) treatment, we found mCAFs and iCAFs exhibit distinct and opposing spatial distribution patterns within the TME, which are associated with patient prognosis. More importantly, we delineated a previously unrecognized spatial capillary–fibroblast signaling axis and uncovered *NOTCH3* as a critical checkpoint governing the functional polarization of mCAFs mediated by endothelial *DLL4*, thereby fortifying the fibrotic barrier and restricting T cell infiltration in HCC. Pharmacological *NOTCH3* inhibition synergized with PD‐1 blockade therapy in both spontaneous and orthotopic implantation HCC murine models, highlighting a promising strategy to overcome immune exclusion and enhance immunotherapy efficacy in HCC by targeting fibrosis.

## RESULTS

### Single‐cell spatial transcriptomic profiles reveal coordinated cellular topography in HCC

**FIGURE 1:**
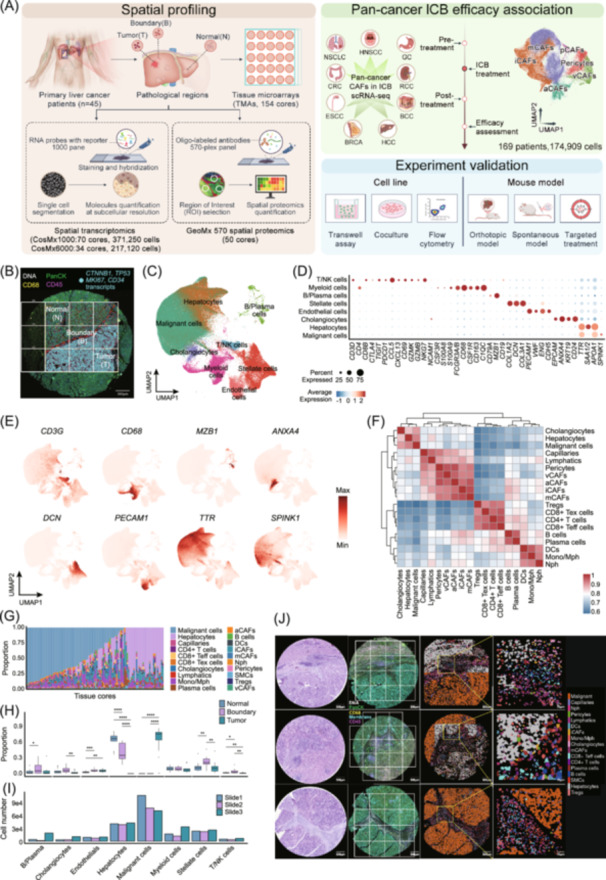
High‐resolution single‐cell spatial analysis uncovers the dynamic landscape of cell populations in HCC. (A) Schematic overview of the study design, sample selection, including spatial transcriptomics (CosMx1000 and CosMx6000), GeoMx spatial proteomics (570‐plex), public scRNA‐seq of CAFs under ICB treatment, and experiment validation. (B) Representative immunofluorescence image of a tissue microarray core, showing Tumor (T), Boundary (B), and Normal (N) regions overlaid all transcripts of *CTNNB1*, *MKI67*, *TP53*, and *CD34* to each Field of View (FOV), in which invasive boundary has been outlined. (C) UMAP visualization of all cells in CosMx1000 data, colored by annotated cell lineages, including Malignant cells, Hepatocytes, Cholangiocytes, Myeloid cells, Stellate cells, Endothelial cells, and B/Plasma cells. (D) Dot plot showing the expression of canonical marker genes across major lineages. (E) Gene expression profiles of canonical marker genes across major cell types from CosMx1000 cohort. (F) Gene expression correlation matrix between cell types from CosMx1000 cohort. (G) Relative proportions of major cell types across all tissue cores. (H) Comparison of cell type proportions among Tumor, Boundary, and Normal regions. The *x*‐coordinate represents each lineage, consistent with the *x*‐axis in (I). (I) Comparison of cell type components among different spatial transcriptomics profiling slides (Slide 1, 2: CosMx1000; Slide 3: CosMx6000). (J) Representative spatial maps of cell types overlaid with H&E and immunofluorescence (IF) staining from selected tissue cores, illustrating the spatial organization of the tumor microenvironment. For statistical significance, *p < 0.05, **p < 0.01, ***p < 0.001, and ****p < 0.0001, ns = not significant. aCAFs, antigen‐presenting CAFs; iCAFs, inflammatory CAFs; ICB, immune checkpoint blockade; mCAFs, matrix‐producing CAFs; Mono/Mph, Monocytes/Macrophages; Nph, Neutrophils; pCAF, proliferative CAFs; vCAFs, vascular CAFs.

To characterize the spatial and phenotypic heterogeneity of cellular components in HCC, we constructed a high‐resolution spatial multi‐omics atlas based on multiple surgically resected specimens. We first generated subcellular‐resolution spatial transcriptomics datasets on two TMA slides using the CosMx™ SMI 1000‐plex panel (CosMx1000) and one TMA slide using the 6000‐plex (CosMx6000) panel. This comprises a total of 104 tissue cores from malignant foci (T), invasive boundaries (B), and adjacent normal parenchyma (N) across 45 HCC patients (Tables S1–S3). In parallel, we performed spatial proteomic profiling targeting 570 proteins on one TMA slide, including 50 tissue cores from a subset of 18 patients within this cohort, covering 95 regions of interest (ROIs) (Table S4). Thirdly, a pan‐cancer scRNA‐seq dataset of CAFs under ICB treatment was collected, in which the detailed timepoint relative to the ICB treatment (Pre or Post treatment) and the clinical efficacy (Responder or Nonresponder) after the ICB has been recorded (Figure 1A, Tables S5, S6). All pathological TMA cores of HCC were checked and selected at a radius of 1 mm by board‐certificate pathologists (Methods). The schematic analysis pipeline was illustrated in Figure S1A,B. To validate the accuracy of pathologists' annotation at the invasive margin region, we labeled all transcripts of *CTNNB1*, *MKI67*, *TP53*, and *CD34* to each tissue core, which were recognized as marker genes involved in HCC development [22, 23]. We found the intensity was enriched in tumor compared with adjacent normal regions apparently, making it possible to outline the invasive front at the FOV image view (Figure 1B).

After a cyclic hybridization and imaging procedure on the tissue slide and cell segmentation, we got a total of 371,250 cells for CosMx1000 cohort and 217,120 cells for CosMx6000 cohort (Figure S1C–E). The median transcript for CosMx1000 is 797 and 829 for CosMx6000 while the median genes detected per cell for CosMx1000 is 227 and 562 for CosMx6000. The median number of cells captured per FOV was comparable between CosMx1000 and CosMx6000 cohorts, with only slight variation observed at the patient level (Figure S1F, Table S7). We totally obtained 774 FOVs (510 nm by 510 nm square) for CosMx1000 cohort, in which 467 belonging to Tumor (T), 53 belonging to Boundary (B) and 254 belonging to Normal (N). For CosMx6000 cohort, there are a total of 402 FOVs, in which 233 belonging to Tumor (T), 49 belonging to Boundary (B), and 120 belonging to Normal (N) (Figure S1G). After standardized single cell data analysis and marker gene‐based cell type annotation, we identified a total of 8 lineages, including Hepatocytes, Malignant, Cholangiocytes, T/NK cells, Stellate cells, Myeloid cells, Endothelials, and B/Plasma cells in CosMx1000 cohort (Figure 1C–E, Figure S1H–J). We further re‐clustered these lineages into subgroups and validated the similarity and specificity between different subtypes by correlation analysis (Figure 1F, Figure S1K,L). The same analysis was also performed on CosMx6000 cohort (Figure S2). We found a heterogeneity in relative proportions of different cell types between different tissue cores (Figure 1G), reflecting the spatial differences in cell composition inter and intra patients. Despite the heterogeneity in relative cell type frequency among different patients and platform, we found stellate cells (also called CAFs) and T/NK cells are significantly enriched in the tumor margin (Figure 1H), indicating CAF aggregation may exclude the infiltration of T cells into TME at the invasive front [24, 25]. The overall cell counts detected by the two platforms are comparable and within the same order of magnitude, and no systematic bias attributable to platform‐specific detection is observed (Figure 1I). Mapping different cell types in situ, we have built a large‐scale single‐cell spatial atlas of HCC with locally matched Immunofluorescence (IF) and H&E staining images (Figure 1J).

### Heterogeneity of malignant cells underlies spatial defined niches to drive tumor progression

**FIGURE 2:**
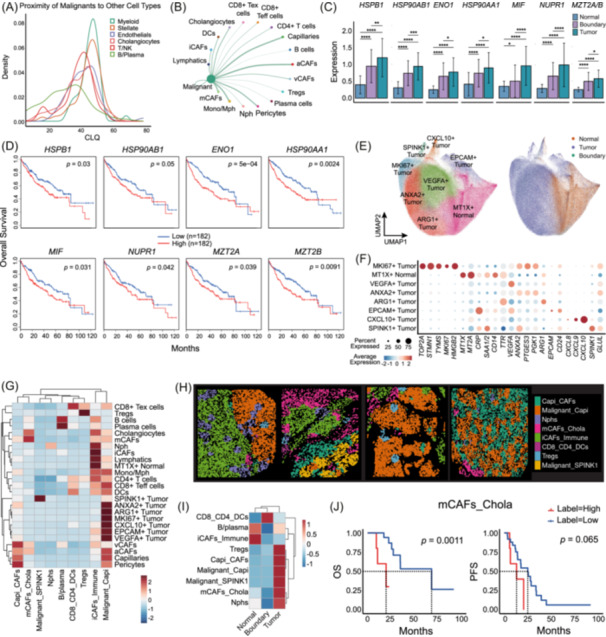
Single‐cell transcriptomics analysis of malignant and hepatocytes throughout the tumor, boundary, and adjacent normal regions. (A) Density of Colocalization Quotient, illustrating the spatial proximity of malignant cells to other lineages. CLQ, a metric measuring the ratio of observed to expected spatial co‐occurrence, where rightward‐shifted peaks indicate a higher frequency of strong colocalization (Methods). (B) Cell–cell communication network analysis of malignant cells to other cell types in CosMx1000 dataset. The analysis incorporated spatial constraints, restricting inferred interactions to biologically feasible distances based on defined interaction ranges for diffusible molecules and contact ranges for contact‐dependent signaling. The thickness of the connecting lines represents the interaction strength (communication probability), calculated based on the expression levels of ligand–receptor pairs. (C) Expression levels of key oncogenic and stress response genes (*HSPB1, HSP90AB1, HSP90AA1, ENO1, MIF, NUPR1, MZT2A/B*) across Normal, Boundary, and Tumor. (D) Kaplan–Meier survival curves for patients with high (red line) and low expression levels (blue line) of key oncogenic and stress response genes. (E) UMAP visualization of all hepatocyte subtypes (including malignant and normal hepatocytes, left) and corresponding Normal, Boundary, and Tumor regions (right). (F) Dot plot showing the expression of canonical marker genes across hepatocyte subtypes. (G) Heatmap of normalized cell type component enrichment in each identified spatial niche. (H) Representative spatial image plot of identified spatial niches. (I) Heatmap of normalized spatial niche component enrichment in each tissue region (Normal, Boundary, and Tumor). (J) Kaplan–Meier survival curves for patients with high and low abundance of mCAFs_Chola niche, indicating a significant association with poor OS and PFS.

To further elucidate the spatial heterogeneity and microenvironmental interactions of malignant cells, we first applied a colocalization quotient algorithm to quantify the spatial proximity between malignant cells and other lineages [26]. Myeloid, stellate, and endothelial cells exhibited strong colocalization with malignant cells, a pattern further supported by ligand–receptor‐based cell–cell communication analysis (Figure 2A,B, Figure S3A,B), suggesting frequent bidirectional crosstalk between these cell types may contribute to tumor progression [27, 28, 29]. Key oncogenic and stress response genes like *HSPB1* [30], *HSP90AB1* [31], *HSP90AA1* [32], *ENO1* [33], *MIF* [34], *NUPR1* [35], and *MZT2A/B*, exhibited a stepwise increase in expression from Normal to Boundary to Tumor, highlighting their spatial enrichment in malignant zones and potential association with poor clinical outcomes (Figure 2C,D, Figure S3C). To resolve tumor cell heterogeneity at finer resolution, we extracted all malignant cells and hepatocytes and reclustered them into seven different tumor clusters and one cluster of normal with transcriptionally distinct phenotype (Figure 2E,F, Table S8). Specifically, *MKI67⁺ Tumor* cells expressed proliferation‐associated genes (*MKI67*, *TOP2A*, *STMN1*, *TYMS*), while *VEGFA⁺ Tumor* cells upregulated genes involved in epithelial/endothelial migration (*VEGFA*, *GLUL*, *COL18A1*, *FASN*, *VHL*). *ANXA2⁺ Tumor* cells were enriched in hypoxia‐related signatures (*PGK1*, *LMNA*, *ENO1*), whereas *ARG1⁺ Tumor* cells expressed metabolism‐related genes (*APOA1*, *APOE*, *HMGCS1*). The *EPCAM⁺ Tumor* subtype was marked by epithelial identity and MHC II‐associated expression (*CD74*, *HLA‐DRA*, *B2M*), and the *CXCL10⁺* Tumor subtype showed activation of interferon signaling (*STAT1*, *CCL5*, *IFITM3*). A *SPINK1⁺* Tumor cluster exhibited upregulation of oxidative stress and apoptosis‐related genes (*SOD2*, *HSPB1*, *PPIA*, *GPX1*), suggesting an intrinsic vulnerability state during tumor progression. In contrast, *MT1X⁺* Normal hepatocytes were restricted to normal regions and enriched in pathways related to leukocyte proliferation and activation, indicating localized immune surveillance (Figure S3D,E). While some subtype‐specific markers were consistently captured, we observed partial differences in the identified tumor cell subtypes between CosMx1000 and CosMx6000 datasets (Figure S4A–F, Table S9), potentially reflecting increased gene coverage and biological heterogeneity rather than solely platform‐dependent bias.

To further explore the spatial organization of malignant cells within the tissue context, we performed spatial neighborhood analysis using the 50 nearest neighboring cells for each cell (Methods) [36]. This revealed nine distinct spatial niches in the CosMx 1000 dataset (Figure 2G,H, Figure S4G,H). Among them, the *Malignant_Capi* niche (malignant cells with capillaries) and *Capi_CAFs* niche (capillaries with diverse CAFs) were consistently identified in both CosMx 1000 and 6000 datasets, suggesting their conserved presence irrespective of panel design (Figure S4I). The *Malignant_SPINK1* niche, composed exclusively of SPINK1⁺ tumor cells, likely reflects a stress‐induced tumor state. Immune‐enriched *iCAFs_Immune* niche containing iCAFs, lymphatic endothelial cells, and multiple immune cell types (neutrophils, monocytes/macrophages, T cells), was primarily localized to adjacent normal tissue, suggesting immune activation outside the tumor core. The *CD8_CD4_DCs* niche was sharply enriched at the invasive boundary (Figure 2I), suggestive of a potential triad of CD8⁺ T cells, CD4⁺ T cells, and dendritic cells involved in antigen presentation and immune response at the invasive front [37, 38]. The *mCAFs_Chola* niche featured co‐localization of mCAFs and cholangiocytes, while its abundance was significantly associated with poor overall survival (OS) and progression‐free survival (PFS) (Figure 2J), implying a tumor‐promoting role via stromal‐epithelial interactions [24]. Additional niches included the *Tregs* niche (Tregs and exhausted CD8⁺ T cells), the *B/Plasma* niche (B and plasma cells), and the *Nphs* niche dominated by neutrophils. Together, these findings reveal coordinated spatial interactions within the TME and suggest a potential malignant cell‐capillary‐mCAF axis, in which tumor‐induced capillaries may be linked with mCAF polarization and contribute to the establishment of an immune‐evasive microenvironment.

### CAF polarization and spatial preference serve as a potential indicator of HCC prognosis

**FIGURE 3:**
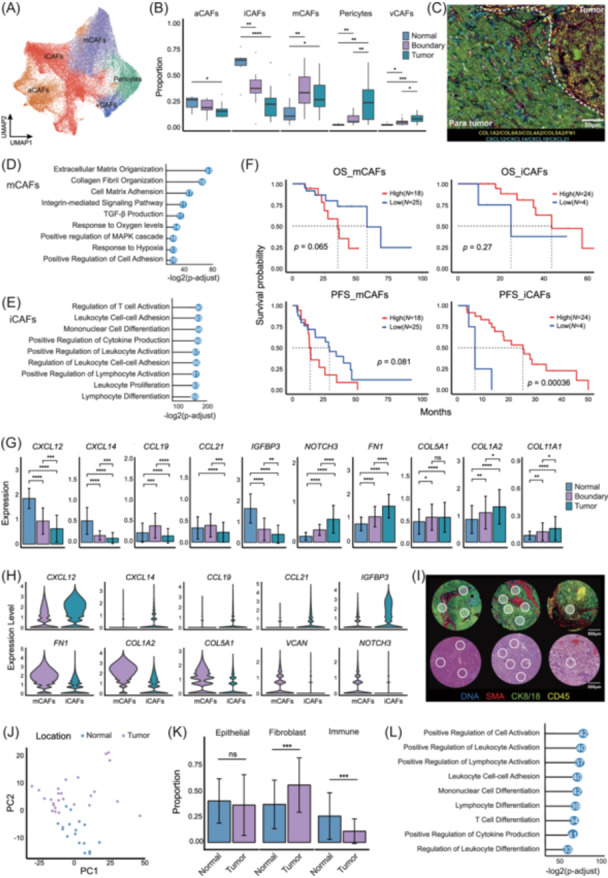
Spatial multi‐omics analysis of CAFs polarization and location. (A) Identification of five distinct CAF subpopulations in spatial data: mCAFs, iCAFs, antigen‐presenting CAFs (aCAFs), vascular CAFs (vCAFs), and pericytes. (B) Proportion of different CAF subtypes across Normal, Boundary, and Tumoral regions. (C) Spatial distribution of mCAF and iCAF markers across Normal, Boundary, and Tumor regions overlaid in IF image, where mCAF‐associated marker genes are labeled in yellow, iCAF‐associated marker genes are labeled in Cyan, and invasive boundary was outlined in White. (D) Gene Ontology (GO) enrichment analysis highlighting functional pathways in mCAFs. (E) GO enrichment analysis highlighting functional pathways in iCAFs. (F) Kaplan–Meier survival curves showing the prognostic significance of mCAF and iCAF proportion. High mCAF infiltration is associated with worse overall survival (OS) and progression‐free survival (PFS). (G) Expression of iCAF‐associated genes (*CXCL12*, *CXCL14*, *CCL19*, *CCL21*) and mCAF‐associated genes (*FN1, COL1A2, COL11A1, COL5A1, NOTCH3*) in CAFs across Normal, Boundary, and Tumoral regions. (H) Violin plot showing the expression of marker genes between iCAFs and mCAFs. (I) Paired IF and H&E images showing the spatial proteomic profiling on FFPE specimens from HCC patients using the GeoMx Immune‐Oncology Protein Panel. A total of 95 regions of interest (ROIs) were selected to represent Tumor, Boundary and Normal tissues. (J) Principal component analysis (PCA) reveals a clear segregation of proteomic profiles between Tumor and Normal regions. (K) Quantitative cell‐type proportion of Epithelial, Fibroblast, and Immune compartment across Tumor and Normal regions. (L) Gene Ontology (GO) enrichment analysis of differentially expressed proteins of ROIs from Normal regions in GeoMx spatial proteomics data. For statistical significance, *p < 0.05, **p < 0.01, ***p < 0.001, and ****p < 0.0001, ns = not significant.

Given the pivotal roles of CAFs in shaping the TME and their prognostic significance in HCC, we conducted an in‐depth spatial transcriptomic analysis to systematically classify CAF subtypes. Six transcriptionally and spatially distinct subpopulations were identified: mCAFs, iCAFs, antigen‐presenting CAFs (aCAFs), vascular CAFs (vCAFs), proliferative CAFs (pCAFs), and pericyte (Figure 3A, Figure S5A,B). To elucidate their spatial dynamics, we performed compartment‐resolved mapping, which revealed that iCAFs were predominantly enriched in adjacent non‐tumoral regions (N), whereas mCAFs preferentially accumulated within tumor cores (T) and invasive margins (B) (Figure 3B,C, Figure S5C). Subsequent Gene Ontology (GO) enrichment analysis highlighted striking functional divergence between subtypes. Specifically, mCAFs were enriched in pathways related to extracellular matrix (ECM) remodeling—including TGF‐β signaling, MAPK activation, and cell adhesion—consistent with a pro‐tumorigenic fibrotic phenotype. In contrast, iCAFs exhibited transcriptional features reminiscent of fibroblastic reticular cells (FRCs) and were strongly linked to adaptive immune responses, such as T cell activation, lymphocyte proliferation, and immune cell chemotaxis (Figure 3D,E, Figure S5D). Clinically, these two CAF subtypes exhibited opposite prognostic associations. High intratumoral mCAF infiltration showed a non‐significant trend towards association with worse OS and PFS (OS: *p* = 0.065, PFS: *p* = 0.081), whereas elevated peritumoral iCAF abundance was linked to favorable clinical outcomes (Figure 3F, Figure S5E). We also analyzed an independent validation cohort using multiplex immunostaining and revealed both mCAF and iCAF proportions with consistent associations with patient outcomes (Figure S5F). Furthermore, molecular profiling in CAFs revealed spatially progressive expression of mCAF‐associated genes (e.g., *FAP*, *FN1*, *COL1A2*, *COL5A1*, *VCAN*, *NOTCH3*) from Normal to Boundary to Tumor regions. Conversely, iCAF‐related gene expression followed a declining gradient across CAFs in these compartments (Figure 3G,H, Figure S5G–I). Collectively, these findings delineate a spatially and functionally dichotomous architecture of CAFs in HCC, underscoring their cooperative yet distinct contributions to TME remodeling and clinical heterogeneity.

To further substantiate the findings obtained from single‐cell spatial transcriptomics, we performed spatial proteomic profiling on FFPE specimens from the same cohort of HCC patients using the GeoMx Immune‐Oncology Protein Panel [39]. A total of 95 ROIs, each approximately 190 nm in radius, were carefully selected to represent malignant foci, invasive margins, and adjacent normal tissues (Figure 3I, Table S4). Principal component analysis (PCA) revealed a clear segregation of proteomic profiles between tumor and peritumoral regions, highlighting distinct molecular landscapes across compartments (Figure 3J). To further dissect the cellular composition within each ROI, we conducted quantitative analysis of IF images, focusing on the relative abundance of epithelial cells (CK8/18⁺), stromal fibroblasts (SMA⁺), and immune cells (CD45⁺) (Methods, Table S10). This spatially resolved analysis revealed compartment‐specific cellular distributions: stromal fibroblasts were significantly enriched in tumor regions, whereas immune cells were predominantly localized in adjacent normal tissues (Figure 3K, Table S10). Notably, proteomic profiling of peritumoral ROIs identified a set of differentially expressed proteins that were significantly associated with signaling pathways involved in T lymphocyte activation and differentiation (Figure 3I). Our spatial multi‐omics correlation analysis revealed distinct functional landscapes. The protein signature correlated with iCAF abundance featured a potent inflammatory secretome (*CXCL10, CCL18, HMGB1*) and immune markers. Mechanistically, iCAF‐enriched regions exhibited constitutive NF‐κB activation, evidenced by enriched IKK components concomitant with negatively correlated IκBα. Conversely, the mCAF‐related proteome was defined by matrix remodeling capabilities (MMP14, CD90) and underpinned by TGF‐β/BMP signaling activation (phosphorylated SMADs), validating a myofibroblastic identity (Figure S5J). Also, as an orthogonal validation, we performed mIHC analyses, which consistently demonstrate a preferential enrichment of iCAFs in the peritumoral regions, whereas mCAFs are predominantly enriched within the tumor core, in agreement with our spatial transcriptomic annotations (Figure S5K). Taken together, these spatial proteomic findings not only corroborate our transcriptomic observations but also reinforce the notion of region‐specific immune and stromal dynamics within HCC microenvironments.

### LSECs‐derived *DLL4* triggers *NOTCH3* activation in mCAFs and ICB resistance

**FIGURE 4:**
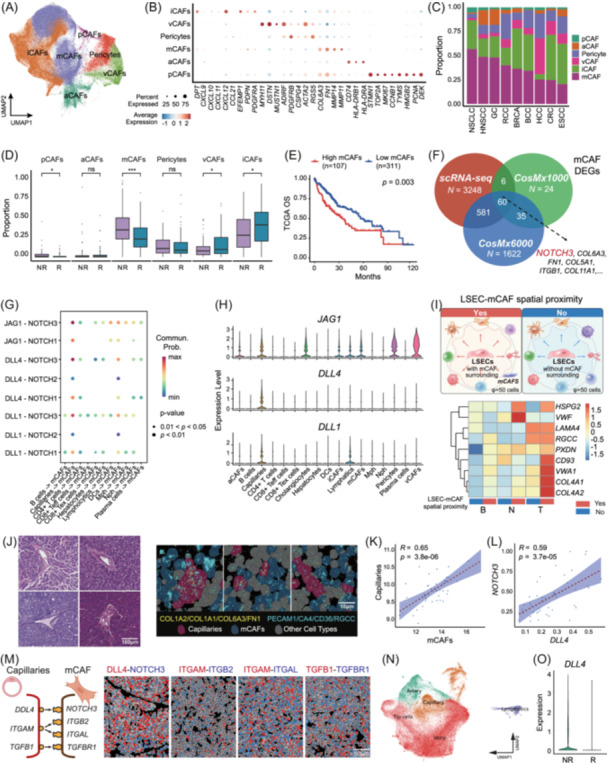
Validation of the clinical potential of CAF polarization based on pan‐cancer scRNA‐seq data under immune checkpoint blockade (ICB) treatment. (A) UMAP plot showing the clustering of different CAF subtypes in pan‐cancer scRNA‐seq data under ICB treatment. (B) Dot plot representing the average expression levels of select marker genes for each CAF subtype. (C) Stacked bar plots showing the distribution of CAF subtypes across various cancer types. (D) Box plots showing the proportion of each CAF subtype in non‐responders (NR) and responders (R) under ICB treatment. (E) Kaplan–Meier survival curves showing the association between mCAF proportion and clinical outcomes (OS) in TCGA‐LIHC data. (F) Venn diagram showing the overlap of mCAF differentially expressed genes (DEGs) from scRNA‐seq and two spatial transcriptomic datasets (CosMx1000 and CosMx6000). The intersection (*n* = 60) is constrained by the CosMx1000 panel size (~1000 genes) but represents 48% (60/125) of the total mCAF DEGs identified in this dataset. *NOTCH3* consistently identified as a robust marker of mCAFs. (G) CellChat ligand‐receptor interaction analysis revealing significant cross talk of the NOTCH signaling pathway between capillaries and mCAFs in CosMx1000 data, primarily mediated by *DLL4/NOTCH3, JAG1/NOTCH3*, and *DLL1/NOTCH3* interactions. (H) Expression levels of *DLL1*, *DLL4*, and *JAG1* across different cell types, showing the sources of these ligands. (I) Schematic diagram for determining whether there is spatial proximity of mCAFs to each liver sinusoidal endothelial cell (LSEC) within 50 spatial neighbors. The below one is Heatmap showing the expression levels of angiogenesis‐related genes in LSECs with (Yes, red) or without (No, blue) spatial proximity to mCAFs. (J) H&E staining and CosMx spatial images illustrated the spatial proximity between capillaries and mCAFs. The H&E image provides histopathological context at the tissue level from an independent cohort, while the IF/ISH panels are representative images illustrating the spatial co‐localization patterns of capillaries and mCAFs identified from the CosMx spatial transcriptomic analysis. (K) Dot plot showing the spatial correlation between mCAF and capillary signatures for each ROI in GeoMx spatial proteomic data. (L) CosMx data revealing the correlation between *DLL4* and *NOTCH3* expression for each FOV. (M) CosMx Spatial imaging data illustrating capillaries–mCAF interactions mediated by different ligands and receptors. (N) UMAP visualization of pan‐cancer endothelial subtypes under ICB treatment. (O) Comparing *DLL4* expression between ICB non‐responders (NR) and responders (R) for capillaries. For statistical significance, *p < 0.05, **p < 0.01, ***p < 0.001, and ****p < 0.0001, ns = not significant.

To comprehensively characterize the diversity of CAF subpopulations at a pan‐cancer level and to elucidate their roles in modulating the immune response, we curated single‐cell RNA sequencing (scRNA‐seq) data from 169 patients who had undergone ICB treatment. Through integrative analysis, we identified 174,909 CAFs, including six major CAF subtypes: mCAFs, iCAFs, vCAFs, aCAFs, pCAFs, and pericytes, mirroring the subtype architecture observed in our spatial‐omics analyses (Figure 4A,B, Table S11). Importantly, the relative abundance of these CAF subtypes varied considerably across different cancer types, underscoring their context‐specific heterogeneity and the potential influence of tumor‐intrinsic and microenvironmental factors (Figure 4C). GO enrichment analysis revealed functional divergence among CAF subtypes, with iCAFs enriched in immune‐regulatory pathways such as T cell recruitment and chemotaxis, while mCAFs were primarily involved in extracellular matrix remodeling and profibrotic signaling; other subtypes exhibited roles in vascular function (vCAFs, pericytes) and cell proliferation (pCAFs) (Figure S6A). To assess the clinical relevance of these subtypes, we evaluated their association with treatment response. Non‐responders exhibited significantly higher proportions of mCAFs, whereas responders were characterized by an enrichment of iCAFs (Figure 4D). Using our spatial transcriptomic profiles as a reference, CIBERSORTx‐based deconvolution [40] of TCGA‐LIHC samples revealed that a higher mCAF proportion is significantly associated with poorer clinical outcomes (Figure 4E). These findings align with our spatial transcriptomics data and reinforce the concept that the functional polarization of CAFs profoundly influences antitumor immunity and clinical responses to ICB therapy.

Given the pivotal role of mCAFs in ECM remodeling and resistance to ICB therapy, we profiled DEGs from scRNA‐seq and two spatial transcriptomic datasets (CosMx1000 and CosMx6000), consistently identifying *NOTCH3* as a robust marker of mCAFs (Figure 4F, Table S11). CellChat ligand–receptor interaction analysis revealed significant activation of the NOTCH signaling pathway between capillaries and mCAFs, primarily mediated by *DLL4/NOTCH3*, *JAG1/NOTCH3*, and *DLL1/NOTCH3* interactions (Figure 4G, Figure S6B,D). Systematic evaluation of ligand distributions across all annotated cell types confirmed a highly restricted expression pattern. Among these, *DLL4* was predominantly expressed in capillaries, showing negligible levels across other major compartments—including fibroblasts (CAFs), immune populations (T cells, B cells, myeloid cells), and epithelial subsets—thereby identifying capillary endothelium as the exclusive source of DLL4 signaling in this niche. (Figure 4H, Figure S6C,E), suggesting that endothelial‐fibroblast crosstalk via the DLL4‐NOTCH3 axis may drive mCAF polarization [3]. Consistent with this, spatial proximity of mCAFs to capillaries (within 80 μm) was associated with increased angiogenic activity, implying a functional interplay between the two cell types (Figure 4I, Figure S6F). This spatial relationship was further corroborated by H&E staining and spatial colocalization analyses (Figure 4J). Also, the proportion of mCAFs surrounding capillaries was found higher in tumor and boundary regions compared with normal regions (Figure S6G). In parallel, GeoMx spatial proteomic profiling demonstrated concordant enrichment of mCAF and capillary signatures, and CosMx data also revealed a strong correlation between *DLL4* and *NOTCH3* expression (Figure 4K–M). Moreover, pan‐cancer analysis of endothelial cells under ICB treatment revealed that *DLL4* expression was significantly elevated in non‐responders (NRs) compared to responders (Figure 4N,O, Figure S6H,I, Table S12), a pattern that was echoed in the TCGA‐LIHC cohort, where both *DLL4* and *NOTCH3* were markedly upregulated in tumor tissues relative to normal liver (Figure S6J). Genes significantly correlated with *NOTCH3* or *DLL4* (Pearson *r* > 0.4, *p* < 0.05) in the TCGA LIHC cohort were primarily involved in ECM organization, cell‐substrate adhesion, and collagen fibril assembly, while *DLL4*‐correlated genes were associated with cell migration and extracellular matrix dynamics (Figure S6K,L, Table S13). Furthermore, transcriptomic profiling of HCC patients receiving adjuvant sorafenib therapy [41] demonstrated significantly higher mCAF infiltration and co‐expression of *NOTCH3* and *DLL4* in NRs compared to responders (Figure S6M). Supporting these findings, analysis of the Fudan HCC cohort [42] revealed that high *DLL4* expression levels were significantly associated with poor clinical outcomes (Figure S6N). Collectively, these findings strongly support a model in which capillaries are linked with mCAF polarization through DLL4‐NOTCH3 signaling.

### mCAF polarization enhances fibrosis and immune exclusion in vitro

**FIGURE 5:**
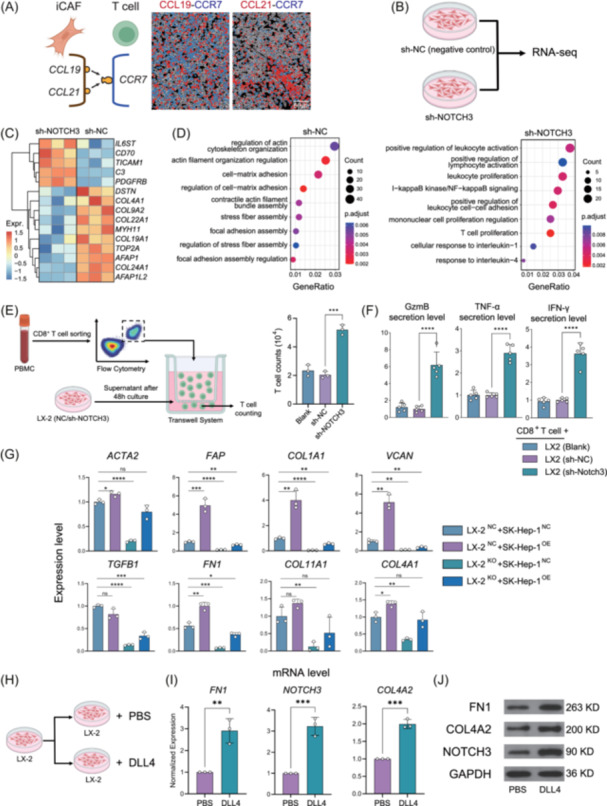
LSECs‐mCAFs crosstalk by DLL4/NOTCH3 axis mediates fibrosis in HCC. (A) Schematic spatial imaging plot of iCAF‐T cells interaction mediated by CCL19/CCL21‐CCR7. (B) Schematic diagram for *NOTCH3* knocking out (KO) experiment on human hepatic stellate cell line LX‐2 cells for bulk RNA‐seq. (C) Up‐regulated and down‐regulated genes between *NOTCH3*‐KO and sh‐NC LX‐2 cell line. (D) Gene Ontology (GO) enrichment terms in *NOTCH3*‐KO and sh‐NC LX‐2 cell line. (E) Schematic diagram for the Transwell experiment and measurement of T cell migration and cytotoxicity. (F) Relative levels of GZMB, TNF‐α, and IFNg detected in the supernatant by ELISA. (G) qPCR results show the expression levels of genes related to fibrotic collagen polarization in different co‐culture groups (SK‐Hep‐1 DLL4‐overexpressing (OE) and SK‐Hep‐1 transfected with empty vector (NC); *NOTCH3*‐knockout (KO) LX‐2 fibroblasts and sh‐NC LX‐2 fibroblasts (NC). (H) Schematic diagram for LX‐2 cells stimulated by DLL4. (I) qPCR analysis compares the mRNA levels of *FN1*, *NOTCH3*, and *COL4A2* between LX‐2 cells with and without DLL4 stimulation. (J) Western blotting compares the protein level of FN1, NOTCH3, and COL4A2 between cells with and without DLL4 stimulation. For statistical significance, *p < 0.05, **p < 0.01, ***p < 0.001, and ****p < 0.0001, ns = not significant.

Using spatial transcriptomics data, we stratified FOVs by immune cell infiltration and identified DEGs in mCAFs and iCAFs associated with infiltration level. Most mCAF signature genes (e.g., *COL4A2*, *COL4A1*, *COL5A3*, *FABP4*, *NOTCH3*) were significantly associated with reduced immune cell infiltration, whereas iCAFs associated genes (e.g., *CXCL9*, *CCL5*, *CXCL13*, and *IGHA1*) correlated with increased infiltration. (Figure 5A). *NOTCH3* knockout in human hepatic stellate cell (HSC) line LX‐2 cells significantly enriched gene signatures related to T cell activation and proliferation (Figure 5B–D, Figure S7A,B). Co‐culture of *NOTCH3*‐deficient LX‐2 cells with CD8⁺ T cells isolated from human peripheral blood mononuclear cells (PBMCs) in a transwell system led to increased T cell migration, with elevated levels of GZMB, TNF‐α, and IFNG detected in the supernatant by ELISA (Figure 5E,F, Figure S7C). Co‐culture experiments using DLL4‐overexpressing [43] or liver sinusoidal endothelial cells (SK‐Hep‐1, Cat# GCL‐0212, RRID: CVCL_0525) transfected with empty vector, and *NOTCH3*‐knockout or sh‐NC LX‐2 fibroblasts confirmed that DLL4‐NOTCH3 signaling facilitates fibrotic collagen polarization (Figure 5G). Compared with PBS‐coated controls, DLL4‐Fc stimulation significantly increased the expression of HES1, a canonical downstream effector of NOTCH signaling. In parallel, the expression of collagen‐related genes, including *FN1*, *NOTCH3*, and *COL4A2* was markedly upregulated (Figure 5H–J, Figure S7D–F). These findings demonstrate that activation of the DLL4‐NOTCH3 axis is associated with mCAF polarization, thereby exacerbating liver fibrosis in the tumor microenvironment.

### mCAFs engage with Mono/Mph to shape immunosuppressive TME

**FIGURE 6:**
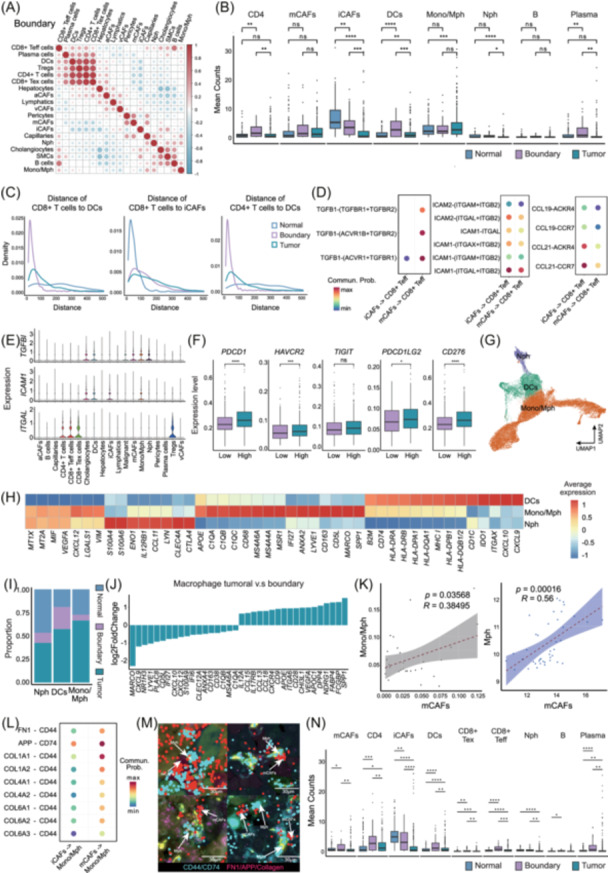
mCAF‐induced polarization shapes an exhaustive TME. (A) Cell type frequency correlation analysis at the invasive tumor boundary shows a coordinated co‐occurrence of CD4⁺ T cells, DCs, and CD8^+^ T cells. (B) Mean counts of different cell types within spatial neighborhoods of 50 surrounding cells centered on CD8⁺ T cells in Normal, Boundary, and Tumor regions. (C) Density plots showing the distance of CD8⁺ T cells to DCs, iCAFs, and mCAFs in Normal, Boundary, and Tumor regions. (D) CellChat analysis between iCAFs/mCAFs and CD8^+^ Teff. (E) Expression of *TGFBI*, *ICAM1*, and *ITGAL* across different cell types. (F) Box plots showing the expression levels of immune checkpoint genes (*PDCD1, HAVCR2, TIGIT, PDCD1LG2, CD276*) in mCAF‐high and mCAF‐low groups. (G) UMAP visualization for different myeloid subtypes. (H) Heatmap showing the expression levels of marker genes across different myeloid subtypes. (I) The stacked barplot of different myeloid cell subtypes across Normal, Boundary, and Tumor regions in CosMx1000 data. (J) Ranked differentially expressed genes (DEGs) for macrophages between tumoral and boundary regions. (K) Scatter plot showing the correlation between mCAF and Mono/Mph proportions in the CosMx 1000 dataset (left). Scatter plot showing the correlation between mCAF and Mono/Mph signature scores in GeoMx 570 dataset (right). (L) CellChat analysis between iCAF/mCAF to Mph. (M) Spatial imaging plot showing the co‐localization between mCAF and macrophages overlaid on the IF image, where mCAF‐associated ligand genes are labeled in red, Mph‐related receptor genes are labeled in Cyan. (N) Mean counts of different cell types within spatial neighborhoods of 50 surrounding cells centered on Mph in Normal, Boundary, and Tumor regions. For statistical significance, *p < 0.05, **p < 0.01, ***p < 0.001, and ****p < 0.0001, ns = not significant.

To investigate the spatial interactions of CD8⁺ T cells, we performed neighborhood analysis and observed that CD4⁺ T cells, and DCs are closely co‐localized with CD8⁺ T cells at the invasive tumor boundary (Figure 6A), suggesting active immune crosstalk at this site (Figure 2I). Notably, iCAFs exhibit preferential spatial colocalization with CD8⁺ T cells in the adjacent non‐tumor region, rather than in the tumor core or boundary (Figure 6B,C, Figure S8A–C), indicating a region‐specific iCAF–immune interaction pattern. Further ligand–receptor analysis revealed that TGF‐β signaling is more prominent between mCAFs and CD8⁺ effector T cells compared to iCAFs (Figure 6D,E, Figure S8D,E), suggesting the immunosuppressive role of mCAFs in CD8⁺ T cell cytotoxicity [44, 45]. In contrast, iCAFs exhibited stronger *ICAM* and *CCL19/CCL21* signaling activity, indicating a potential role in recruiting and retaining effector T cells within the TME [46, 47]. These findings highlight the distinct immunoregulatory roles of CAF subtypes in shaping T cell function and distribution. Furthermore, we found immune checkpoint genes such as *PDCD1*, *HAVCR2*, *TIGIT*, *CD276*, and *PDCD1LG2* were significantly upregulated in the mCAF‐high group (Figure 6F).

Besides T/NK cells, we found myeloid cells, including DCs, Mono/Mph, and Nphs exhibited distinct spatial distribution patterns across TME. Mono/Mph were predominantly enriched in the tumor core, whereas Nphs were more abundant in adjacent normal tissues. DCs were mainly located at the invasive boundary, consistent with previous findings (Figures 2, 6, Figure S8F–K). A series of M2‐associated genes exhibited a stepwise increase toward the tumor core, with *SPP1* being the most enriched. This aligns with previous studies indicating that SPP1⁺ macrophages contribute to T cell exhaustion (Figure 6J) [48, 49, 50, 51]. Consistently, both CosMx SMI spatial transcriptomics and GeoMx spatial proteomics data support a strong correlation between mCAF accumulation and macrophage recruitment, indicating that a pro‐fibrotic TME driven by mCAFs further promote immune evasion (Figure 6K). Furthermore, collagens, APP, and FN1 expressed on mCAFs showed a strong interaction with Mono/Mph via CD44 and CD74, which may trigger signaling pathways that contribute to immune exhaustion (Figure 6L,M) [52, 53]. Additionally, we found an increased local co‐localization or spatial proximity between mCAFs and macrophages at the invasive front, suggesting these pro‐tumorigenic interactions for immunosuppression (Figure 6N).

### *NOTCH3* inhibition augments anti‐PD‐1 efficacy by enhancing immune activation in vivo

**FIGURE 7:**
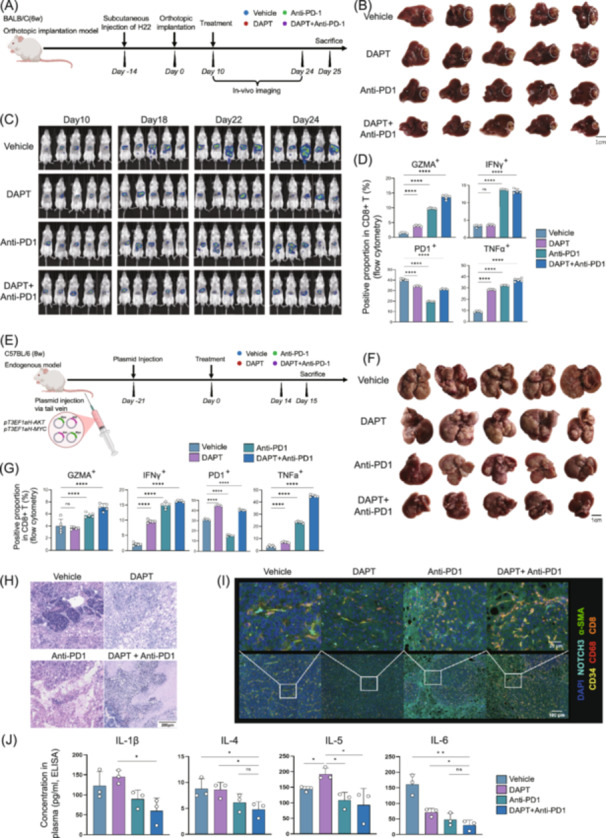
Experiment validation for NOTCH‐targeted liver cancer treatment in mouse models. (A) Schematic diagram for establishing mouse liver cancer orthotopic implantation model, and applying treatment and in vivo imaging on the model (DAPT: a small molecule NOTCH inhibitor). (B) Representative images of tumor size among different treatment groups using orthotopic implantation model. (C) In vivo live imaging on different time points, comparing fluorescence signals among different treatment groups using orthotopic implantation model. (D) The proportion of GZMA^+^, IFNγ^+^, PD1^+^, and TNF‐α^+^ cells in CD8^+^ T cells, comparing among different treatment groups using orthotopic implantation model. (E) Schematic diagram for establishing endogenous liver cancer mouse model and treatment. (F) Representative images of liver size among different treatment groups in the endogenous model. (G) The proportion of GZMA^+^, IFNγ^+^, PD1^+^, and TNF‐α^+^ cells in CD8^+^ T cells from the endogenous model, comparing among different treatment groups in the endogenous model. (H, I) HE and multiplex immunohistochemical staining images of different treatment groups in endogenous model. (J) Concentration of IL‐1b, IL‐4, IL‐5, and IL‐6 in plasma of different treatment groups in the endogenous model. For statistical significance, *p < 0.05, **p < 0.01, ***p < 0.001, and ****p < 0.0001, ns = not significant.

Given that our spatial multi‐omics analysis identified DLL4‐NOTCH3 signaling as a central axis in CAF polarization, we next sought to functionally perturb NOTCH signaling. We applied DAPT, a γ‐secretase inhibitor that broadly suppresses NOTCH receptor activation, as a pharmacological approach to attenuate NOTCH signaling. Although DAPT is not specific to *NOTCH3*, the consistent enrichment of *NOTCH3* in CAFs suggests that the observed effects are at least partially mediated through *NOTCH3*. To evaluate the role of NOTCH pathway in modulating response to ICB, we established an orthotopic HCC implantation model (Figure 7A). After 14 days of tumor establishment subcutaneously, tumors were orthotopically implanted into the mouse liver. After 10 days, animals were randomly assigned to receive Vehicle, *NOTCH* inhibitor DAPT, Anti‐PD1 antibody, or the combination therapy (DAPT+Anti‐PD1). We found that the combination treatment markedly reduced overall tumor burden in orthotopic HCC mouse model (Figure 7B,C). Flow cytometry analysis revealed that tumor infiltrating CD8⁺ T cells exhibited significantly enhanced cytotoxic activity following combination therapy (Figure 7D), accompanied by extensive immune cell infiltration and exacerbated tumor necrosis (Figure S9A–C). A parallel HCC endogenously arising mouse model via tail vein injection of a combination of Myc and Akt encoding plasmids was also performed to validate the synergistic effects of *NOTCH3* inhibition and immunotherapy (Figure 7E). We found monotherapy with either DAPT or Anti‐PD‐1 modestly reduced tumor growth compared to vehicle. Notably, combined treatment with DAPT and anti‐PD‐1 led to the most significant suppression of tumor progression (Figure 7F, Figure S9D–F). Flow cytometry and histological analysis further revealed a marked increased immune cell infiltration, upregulation of cytotoxic activity of CD8^+^ T cells in the TME, reduction in hepatic fibrosis, and elevated necrosis in the combination group compared to monotherapies or vehicle (Figure 7G–I). Further ELISA analysis of mouse plasma from the two animal models revealed that levels of cytokines, including IL‐1β, IL‐4, IL‐5, and IL‐6, were all reduced in the combination treatment group, shifting the immunosuppressive state (Figure 7J). Together, these results show that *NOTCH3* inhibition enhances anti‐PD‐1 efficacy by boosting antitumor immunity and reversing immune suppression.

## DISCUSSION

HCC is frequently characterized by abnormal fibrosis and immune exclusion, both of which contribute significantly to disease progression and immunotherapy resistance [53, 54]. In this study, we leveraged high‐plex single‐cell resolution spatial transcriptomics, integrated with spatial proteomics and pan‐cancer scRNA‐seq data of CAFs under ICB treatment to delineate the spatial architecture, functional polarization, and clinical implications of CAFs in HCC. This high‐dimensional atlas provides key insights into the cellular and molecular basis of immune suppression in HCC. We found that capillary‐derived *DLL4* activates *NOTCH3* to drive mCAF polarization, forming an immune‐restrictive niche that limits T cell infiltration and promotes fibrosis. Targeting *NOTCH3* enhances tumor sensitivity to ICB therapy in vivo, supporting its potential as a therapeutic target to reduce fibrosis and improve efficacy of immunotherapy in HCC.

The inclusion of pan‐cancer CAF analyses was motivated by our previous work in establishing the scICB database [55], a curated pan‐cancer single‐cell atlas that systematically integrates single‐cell RNA‐seq data from patients receiving immune checkpoint blockade therapy, together with well‐annotated treatment time points and clinical outcome information. This resource provides a unique framework to investigate cell states from diverse cell types in the context of immunotherapy response across tumor types. In the present study, we leveraged this pan‐cancer dataset to explore whether CAF phenotypic polarization, particularly mCAFs and iCAFs, shows conserved associations with immunotherapy response beyond HCC. Specifically, CAF subtypes were identified and annotated using consistent marker‐based and functional criteria, and their transcriptional features were analyzed in relation to treatment response and clinical outcomes. This analysis was designed to complement, rather than replace, the HCC‐focused spatial multi‐omics results, providing an external and independent context in which to evaluate the potential immunoregulatory relevance of distinct CAF states.

The altered composition and architecture of the ECM driven by mCAFs polarization not only provide structural support for tumor cells growth, but also act as a physical barrier to hinder anti‐tumor immune cell infiltration into the tumor [56]. Besides, fibrotic TME fosters an immunosuppressive milieu by exhausting T cells and recruiting immunosuppressive cells like regulatory T cells, myeloid‐derived suppressors cells, and neutrophils [57, 58]. Consequently, targeting the fibrotic TME has recently emerged as a promising strategy to enhance immunotherapy efficacy [59, 60]. Preclinical studies have shown that the combination of anti‐fibrotic agents with immunotherapy exhibits synergistic efficacy, providing a compelling rationale for further research [61, 62]. Elucidating the mechanisms linking anti‐fibrotic and immunotherapeutic agents will aid in predictive biomarker identification and guiding precise patient selection.

In the TME, inflammatory CAFs are considered the major immune‐modulating subsets and characterized by elevated production of inflammatory cytokines such as IL‐6 and LIF and low levels of α‐SMA [63]. Unlike mCAFs, which are typically located near tumor foci, iCAFs are predominantly found in more distal tumor regions [58, 64]. Previous studies suggested iCAFs are closely associated with immune evasion and tumor progression, and actively contribute to fibrotic remodeling within the tumor microenvironment [17, 65]. However, the iCAFs identified in our pan‐cancer scRNA‐seq integrative analysis closely resemble FRCs, characterized by high expression of *CCL19*, *CCL21*, *CXCL13*, and *CXCL14*, indicating the role in anti‐tumor immune response. FRCs have been reported to play critical roles in T cell recruitment, lymph node remodeling, and creating specialized niches that support immune activation [66, 67, 68]. CCL19⁺ iCAFs have been shown to suppress colorectal cancer liver metastasis by recruiting B cells to promote the formation of tertiary lymphoid structures [69]. These observations underscore the context‐dependent roles of iCAFs, contributing both tumor‐promoting and suppressing immune response.

Compared with previous studies that have broadly characterized CAFs as key modulators of fibrosis and immune regulation in HCC, our work provides a more spatially resolved and mechanistically integrated perspective on CAF heterogeneity and function. By combining high‐resolution spatial multi‐omics profiling of HCC with pan‐cancer single‐cell transcriptomic data from ICB cohorts, we identified two distinct CAF polarization states mCAFs and iCAFs, and uncovered their differential associations with patient prognosis and therapeutic response. These findings offer direct multi‐omics evidence that targeting collagen‐producing CAF polarization may represent an effective strategy to remodel the fibrotic stroma and enhance immune responsiveness. In the signature of mCAF polarization, we identified key effectors executing this multidimensional suppression. Beyond the structural barrier formed by *COL11A1*, *VCAN*, and *FN1*, we observed high expression of the immune checkpoint *CD276* (B7‐H3) and the immunosuppressive lectin *LGALS1* (Galectin‐1), suggesting that mCAFs actively dampen T cell function via direct signaling rather than acting merely as passive shields. Furthermore, the upregulation of glycolytic enzymes such as *ENO1* and *PGK1* implies a metabolic reprogramming in mCAFs, which may contribute to a nutrient‐deprived, hypoxic niche that further restricts effector T cell viability. As the most important signaling, we identified *NOTCH3* as a key receptor driving mCAF polarization, activated by the endothelial ligand *DLL4*, thereby delineating a previously unrecognized capillary–fibroblast signaling axis that links vascular cues to stromal remodeling and immune exclusion.

Our prioritization of the DLL4‐NOTCH3 signaling axis is grounded in a multi‐layered convergence of spatial, transcriptomic, and mechanistic evidence, driven by a strategic focus on identifying actionable signaling receptors rather than generic markers. Specifically, we prioritized *NOTCH3* from our candidate list not only due to its robust enrichment in mCAFs across independent cohorts (including CosMx and pan‐cancer datasets) but also as a critical surface receptor capable of mediating external signals, a prerequisite for designing targeted interventions. This receptor‐centric selection was further validated by our spatial ligand–receptor analysis, which revealed a distinct physical proximity between DLL4^+^ endothelial niches and NOTCH3^+^ mCAFs, suggesting a spatially privileged signaling axis rather than a diffuse interaction. Furthermore, given the phenotypic alignment between mCAFs and activated HSCs, the established involvement of NOTCH signaling in liver fibrosis provides a strong biological precedent. Our discovery extends prior observations of *NOTCH3* as a regulator of hepatic fibrosis, revealing its additional role in orchestrating CAF collagen polarization within the tumor microenvironment. Targeting this DLL4‐NOTCH3 pathway may thus provide a promising avenue for combinatorial strategies integrating antifibrotic and immunotherapeutic approaches.

Given the pivotal role of NOTCH signaling in shaping CAF subtypes, therapeutic inhibition of this pathway has been actively explored in clinical settings. In PDAC, γ‐secretase inhibitors MK‐0752 plus gemcitabine yielded encouraging responses [70]. Selective γ‐secretase inhibitors BMS‐986115, LY900009, and nirogacestat have demonstrated safety, sustained target engagement, and clinical benefit in phase I–III trials for pancreatic, breast, and desmoid tumors [71, 72, 73]. However, no analogous NOTCH‐targeted clinical trials have been initiated for liver cancer. Our pre‐clinical findings highlight *NOTCH3* as a prominent candidate orchestrating stromal remodeling and immune evasion in HCC. While γ‐secretase inhibition by DAPT provided supportive in vivo evidence by attenuating fibrosis and enhancing immune infiltration, it is important to acknowledge the limitations of this approach. DAPT is not a *NOTCH3*‐specific inhibitor but rather a pan‐NOTCH inhibitor that also interferes with the processing of multiple non‐NOTCH substrates, and thus its effects may not exclusively reflect *NOTCH3* blockade. Nevertheless, the convergence of multiple layers of evidence strengthens our conclusion. First, scRNA‐seq and spatial transcriptomic analyses consistently identified *NOTCH3* as a robust marker of mCAFs, closely associated with stromal activation signatures. Second, in vitro data confirmed the correlation between *NOTCH3* expression and fibrotic features along with immunosuppressive phenotypes. Third, the phenotypic changes observed upon γ‐secretase inhibition were directionally consistent with the predicted role of *NOTCH3* derived from multi‐omics analyses. Future work using more specific perturbation tools, such as *NOTCH3*‐selective inhibitors, genetic knockdown, or conditional knockout models, will be necessary to definitively establish causal roles of *NOTCH3* in CAF biology and to assess its translational potential as a therapeutic target.

Our spatially resolved omics analysis extends current insights into HCC stromal heterogeneity. We validated aggressive matrix‐remodeling mCAFs and immunomodulatory iCAFs [74], specifically a CCL19^+^ subset resembling FRCs observed in chronic inflammation [75, 76]. Crucially, our application of CosMx SMI provides a distinct technological advantage over previous spot‐based spatial transcriptomics (e.g., Visium). By resolving cellular composition at single‐cell resolution rather than regional mixtures, we precisely mapped the spatial segregation of CAF niches, which serve as structural determinants of “immune‐hot” versus “immune‐cold” microenvironments [77]. Mechanistically, we uncover a novel endothelial‐CAF axis driving mCAF polarization, distinct from canonical TGF‐β signaling. We identified endothelial‐derived *DLL4* as the specific ligand activating *NOTCH3* in fibroblasts to enforce immune exclusion. This aligns with recent single‐cell analyses [78] but uniquely pinpoints liver sinusoidal endothelial cells as the upstream signal source. Consequently, we validated that targeting this DLL4‐NOTCH3 axis offers a strategic therapeutic avenue to normalize the stroma and potentiate immune checkpoint blockade.

To precisely define the cellular ontology of these therapeutic targets, lineage tracing technology serves as the gold standard in murine models [76, 77]. Zhu et al. established the cellular origin of CD36^+^ CAFs in murine HCC tumors by using a lineage‐tracing strategy [78]. Although definitive lineage tracing is restricted in human samples, our transcriptomic profiling indicates that mCAFs most closely resemble activated HSCs rather than their quiescent counterparts. Specifically, the mCAF population expresses canonical myofibroblastic markers (e.g., *COL1A1*, *ACTA2*) while lacking quiescent signatures (e.g., *RBP1*, *LRAT*). However, these cells appear to be more than simply activated HSCs; their distinct angiogenic and immunomodulatory features suggest a tumor‐educated state. Future studies elucidating the precise trajectory from quiescent HSCs to this tumor‐conditioned phenotype will be critical for distinguishing lineage‐dependent mechanisms from microenvironment‐driven plasticity, ultimately refining therapeutic targeting by distinguishing the lineage‐dependent versus microenvironment‐driven mechanisms that underlie CAF heterogeneity in HCC. Our findings here provide the necessary molecular framework to guide these future lineage‐tracing efforts, serving as a rationale to validate the specific dynamic origins of mCAFs.

Technically, our study leverages a dual‐panel strategy reflecting the rapid evolution of spatial transcriptomics. While the initial 1000‐gene panel effectively captured core tumor‐microenvironment interactions, the subsequent application of the 6000‐gene panel provided rigorous validation of these findings. This comparison not only confirms the robustness of our identified spatial domains but also offers a timely technical benchmark for the field, demonstrating that key biological patterns remain consistent and reproducible across varying depths of targeted gene profiling.

Integrating spatial multi‐omics with other diagnostic modalities holds significant promise for precision medicine. While spatial assays define localized stromal or immune niches, combining them with liquid biopsy could enable non‐invasive, longitudinal monitoring of stromal remodeling and therapy response. Additionally, emerging long‐read sequencing technologies like Oxford Nanopore could further resolve isoform‐specific variances within these spatial niches, refining therapeutic targeting. Standard imaged‐based spatial transcriptomics like CosMx relies on probe hybridization, which may miss complex splicing events. Combining this with Nanopore (ONT) long‐read sequencing could resolve isoform‐specific expression, for example, specific *NOTCH3* splice variants within different spatial niches. Recent advances in “Spatial Isoform Transcriptomics” have demonstrated the ability to map full‐length isoform diversity to specific tissue structures [79], which is crucial for designing highly specific inhibitors that target pathogenic isoforms while sparing physiological ones. Regarding clinical feasibility, although high‐plex spatial assays currently entail higher costs and longer turnaround times, their unique value lies in identifying spatial biomarkers, such as capillary‐mCAF niche, which traditional bulk sequencing inherently misses. Given the high cost of ICB therapy, precise patient stratification using these spatial signatures offers a favorable cost‐benefit ratio by avoiding ineffective treatments. Future translational efforts may focus on developing simplified, cost‐effective diagnostic panels based on these high‐plex discoveries for routine clinical use.

Image‐based spatial transcriptomics inherently relies on optical segmentation and spatial proximity to assign transcripts to individual cells, which may introduce partial signal contamination, particularly in regions with high cellular density or complex tissue architecture. This limitation is especially relevant at tissue interfaces and invasive fronts, where tightly packed or interdigitated cell populations increase the likelihood of transcript spillover across neighboring cell boundaries. Firstly, more concise cell segmentation and annotation guided by high‐resolution immunofluorescence imaging and pathologist‐verified histological landmarks are needed to ensure accurate cellular identity. Secondly, stringent quality control filters were also needed to exclude cells with low transcript counts or ambiguous segmentation. Third, all spatial analyses performed at the population level rather than relying on single‐gene expression in individual cells may reduce the impact of sporadic signal leakage. Finally, key spatial patterns were validated using orthogonal approaches, including marker co‐localization, regional enrichment analyses, and consistency across independent samples. Together, these measures minimize the influence of partial signal contamination and support the robustness of the spatial conclusions drawn in this study.

## CONCLUSION

In conclusion, our study underscores the critical role of CAF polarization in shaping the immune landscape of HCC and identifies NOTCH pathway, especially DLL4‐NOTCH3, as a novel therapeutic axis to target. By disrupting the fibrotic niche and enhancing immune infiltration, targeting CAFs could pave the way for more effective combination therapies in HCC, ultimately improving patient outcomes.

## METHODS

### Human subjects and sample collection

Human FFPE tissue samples from surgically resected primary HCC patients were collected from the Department of Pathology at Beijing Tsinghua Changgung Hospital retrospectively. Ethical approval was granted by the Ethics Committee of Beijing Tsinghua Changgung Hospital, Tsinghua University (Approval No. 23642‐0‐01), and written informed consent was obtained from all participants.

### Pathological sample selection for spatial multi‐omics profiling

To increase throughput, 104 tissue cores were assembled into three TMA slides for spatial transcriptomics and 50 cores into one TMA slide for spatial proteomics. Board‐certified pathologists selected representative malignant and adjacent normal regions from H&E sections, with up to three cores per patient.

### CosMx™ spatial molecular imaging (SMI) profiling

Slides were processed with CosMx™ Human Universal Cell Characterization RNA Panels (1000‐plex and 6000‐plex), followed by deparaffinization, antigen retrieval, digestion, fiducial labeling, hybridization, and loading onto the SMI instrument. Iterative hybridization‐imaging cycles (16 pools for 1000‐plex, 27 for 6000‐plex) enabled multiplexed RNA detection, followed by antibody and DAPI staining for morphological imaging and cell segmentation.

### High‐plex spatial proteomics via GeoMx DSP protein assay

High‐plex spatial proteomics was performed with GeoMx DSP platform (Bruker). FFPE slides were deparaffinized, antigen‐retrieved, blocked, and incubated with oligo‐tagged antibodies and morphology markers. Ninety‐five CAF‐enriched ROIs were annotated per core, and antibody tags were UV‐released, amplified, and sequenced (Illumina NovaSeq). Sequencing counts were mapped to spatial coordinates to generate high‐resolution proteomic maps.

### Preprocessing, integration, and dimension reduction of CosMx SMI data

Single‐cell spatial transcriptomic data were processed using the Seurat package (v5.0.1). Library size normalization, SCTransform, and patient‐level integration were performed with IntegrateLayers for batch correction while preserving spatial context. The integrated data were scaled, reduced with PCA and UMAP, and used for clustering, differential expression, and spatial interaction analyses. Cell type annotation was performed based on canonical marker genes (Supplemental Methods) and cluster‐specific highly expressed genes.

### GeoMx DSP data processing and analysis

Raw data were trimmed, merged, and aligned; UMIs were used to remove PCR duplicates to generate accurate counts. Protein expression was aggregated per sample, filtered for outliers, and normalized. Proteins with SNR < 3 were excluded, and differential expression was analyzed using *t*‐tests in R.

### Pan‐cancer integration of CAFs from scRNA‐seq datasets under ICB treatment

Single‐cell RNA‐seq datasets related to ICB therapy were retrieved from scICB database (Ji et al. n.d.). CAFs from tumor samples were merged, normalized, and integrated using Seurat (v5.0.3) with RPCA for batch correction. UMAP‐based clustering identified subpopulations, DEGs were detected (Wilcoxon test), and cell types were annotated using canonical markers (Supplemental Methods).

### HCC mouse model construction

For the implantation model, H22 tumor fragments were implanted into the liver of BALB/c mice, while in the endogenous model, C57BL/6 mice received hydrodynamic tail vein injection of Akt, Myc, and SB plasmids [80]. After tumor establishment, mice were randomized into four groups: Vehicle, DAPT (10 mg/kg, i.p., daily), anti‐PD‐1 (10 mg/kg, i.p., every 3 days), or combination therapy. Following 14 days of treatment, tumors were harvested for weight measurement and further analysis.

### Flow cytometry analysis

Liver tumors were dissociated into single‐cell suspensions, filtered, and incubated with Fc Block before surface and intracellular antibody staining using standard FACS buffer. Samples were analyzed on a BD FACSLyric™ flow cytometer to characterize immune cell populations.

### Cytokine antibody array

Plasma cytokine profiles were assessed using the RayBio® Mouse Cytokine Antibody Array (QAM‐TH17‐1‐1) following the manufacturer's protocol. Fluorescence signals for 18 cytokines were detected with an Axon GenePix 4000B scanner and quantified using GenePix Pro software.

### Statistical analysis

All statistical analyses were performed using R software (v4.2.0) and GraphPad Prism (v9.0). Data are presented as mean ± standard deviation (SD) unless otherwise indicated. Comparisons between two groups were conducted using two‐tailed Student's *t*‐tests for normally distributed data or Wilcoxon rank‐sum tests for non‐parametric data. For comparisons involving more than two groups, predefined pairwise comparisons were conducted using two‐tailed Student's *t* tests rather than overall one‐way ANOVA, as the analyses were hypothesis‐driven and not intended for global group difference testing. For box‐and‐whisker plots, the center line indicates the median, the box denotes the interquartile range (IQR), and whiskers represent the range or 1.5 × IQR. For correlation analyses, correlation coefficients were computed using Pearson's correlation for approximately linear relationships with continuous variables; Spearman's rank correlation was used when monotonic but non‐linear relationships were anticipated or when robustness to outliers was required. Survival analyses were performed using Kaplan–Meier curves and log‐rank tests. Cox proportional hazards regression analyses were not performed; therefore, no multivariable adjustment for clinical covariates (e.g., age, tumor stage) was applied. Differential expression analyses in single‐cell and spatial transcriptomics datasets were carried out with Seurat's built‐in statistical framework, and *p* values were adjusted for multiple testing using the Benjamini–Hochberg false discovery rate method. Statistical significance was defined as *p* < 0.05 after correction when applicable; significance is denoted as *p < 0.05, ** *p* < 0.01, ***p < 0.001, and ****p < 0.0001.

## AUTHOR CONTRIBUTIONS

**Fansen Ji**: Conceptualization; methodology; data acquisition; data analysis; figure preparation; manuscript writing. **Haochen Li**: Data acquisition; data analysis; figure preparation. **Qi Wang**: Data acquisition; figure preparation. **Jiawei Zhang**: Data analysis; interpretation. **Ying Xiao**: Data acquisition. **Huan Li**: Data acquisition. **Hao Liu**: Data acquisition; **Tanqing Long**: Data acquisition. **Boyang Wu**: Data analysis and interpretation. **Hao Chen**: Data analysis and interpretation. **Haoming Xia**: Data analysis and interpretation. **Xinquan Liu:** Data acquisition. **Chuanrui Xu:** Study supervision. **Yibo Gao**: Data analysis; study supervision. **Bingjun Tang**: Data acquisition; funding acquisition. **Juan Liu**: Methodology development; funding acquisition; study supervision. **Shizhong Yang**: Conception; methodology; funding acquisition; study supervision. **Jiahong Dong**: Conception; methodology; funding acquisition; study supervision. All authors have read the final manuscript and approved it for publication.

## CONFLICT OF INTEREST STATEMENT

The authors declare no conflicts of interest.

## ETHICS STATEMENT

The study protocol was approved by the Ethics Committee of Beijing Tsinghua Changgung Hospital, Tsinghua University (NO. 23642‐0‐02). The study conformed to the principles of the Helsinki Declaration.

## Supporting information


**Figure S1.** Overview of spatial transcriptomic and proteomic profiling in HCC.
**Figure S2.** Overview of CosMx 6000 spatial transcriptomic data.
**Figure S3.** Additional analysis on spatial heterogeneity and interactions of tumor cells.
**Figure S4.** Analysis on spatial heterogeneity and interactions of tumor cells in CosMx6000 data.
**Figure S5.** Additional analysis of CAFs polarization and spatial preference in HCC.
**Figure S6.** Detailed analysis of LSECs‐mCAFs crosstalk by DLL4/NOTCH3 axis.
**Figure S7.** Experiment validation of LSECs‐mCAFs crosstalk by DLL4/NOTCH3 axis.
**Figure S8.** Comprehensive analysis of immune cell distribution and interactions with CAFs in the TME.
**Figure S9.** Additional results of NOTCH‐targeted treatment in mouse models.

**Table S1.** Patient information for spatial multiomics anlaysis in this study.
**Table S2.** CosMx™ Universal Cell Characterization Panel (1000 plex) ‐ Gene and Probe Details.
**Table S3.** CosMx™ Human 6k Discovery Panel (6000 plex) ‐ Gene and Probe Details.
**Table S4.** GeoMx® IO Proteome Atlas ‐ Protein Details.
**Table S5.** Patient information of public pan‐cancer scRNA‐seq data under ICB treatment.
**Table S6.** Response definition of public pan‐cancer scRNA‐seq data under ICB treatment.
**Table S7.** Field of Views (FOVs) information.
**Table S8**. Signature genes of malignant cells in CosMx1000 dataset.
**Table S9.** Signature genes of malignant cells in CosMx6000 dataset.
**Table S10.** Relative proportion of different compartments in GeoMx data.
**Table S11.** Signature genes of mCAFs and iCAFs in spatial transcriptomics and scRNA‐seq data.
**Table S12.** Endothelial marker genes of pan‐cancer scRNA‐seq data under ICB treatment.
**Table S13.** Signature genes that are significantly correlated with NOTCH3 and DLL4 in TCGA data.
**Table S14.** PCR primer information.

## Data Availability

The CosMx1000, CosMx6000, and GeoMx data are available from the Zenodo database (https://zenodo.org/records/16912058). RNA‐seq data in knocking‐out cell lines are available in Genome Sequence Archive (GSA) with accession codes HRA012256 (https://ngdc.cncb.ac.cn/gsa-human/browse/HRA012256). The sample information is listed in online Supplemental Table S1. Due to ethical and legal restrictions, deidentified individual participant data and the data dictionary cannot be made publicly available. All data are available upon request to the corresponding author and subject to local rules and regulations. Public scRNA‐seq data are available in Gene Expression Omnibus (GEO) and European Nucleotide Archive (ENA) under accession numbers (GSE205506, GSE179994, GSE232240, GSE207422, GSE145281, GSE229353, GSE123813, GSE169246, GSE120575, GSE206325, GSE200996, GSE203115, GSE212217, GSE164237, GSE246613, GSE236581, PRJEB40416), which could be referenced in Table S6. The scripts used are saved in GitHub: https://github.com/JiFansen/NOTCH3_CosMx. Supplementary materials (figures, tables, graphical abstract, slides, videos, Chinese translated version, and update materials) may be found in the online DOI or iMeta Science http://www.imeta.science/.
