# Supplementary material for: Spatial multi‐omics identifies a NOTCH3‐mediated capillary–mCAF crosstalk driving immune exclusion in hepatocellular carcinoma
Source: Imeta. 2026 Mar 17;5(2):e70117. doi: 10.1002/imt2.70117 (PMC13147954; doi:10.1002/imt2.70117)
Supplement: Supplementary file 1 — Figure S1. Overview of spatial transcriptomic and proteomic profiling in HCC. Figure S2. Overview of CosMx 6000 spatial transcriptomic data. Figure S3. Additional analysis on spatial heterogeneity and interactions of tumor cells. Figure S4. Analysis on spatial heterogeneity and interactions of tumor cells in CosMx6000 data. Figure S5. Additional analysis of CAFs polarization and spatial preference in HCC. Figure S6. Detailed analysis of LSECs‐mCAFs crosstalk by DLL4/NOTCH3 axis. Figure S7. Experiment validation of LSECs‐mCAFs crosstalk by DLL4/NOTCH3 axis. Figure S8. Comprehensive analysis of immune cell distribution and interactions with CAFs in the TME. Figure S9. Additional results of NOTCH‐targeted treatment in mouse models. [file IMT2-5-e70117-s002.docx]

**Spatial Multi-Omics Identifies a NOTCH3-Mediated Capillary–mCAF Crosstalk Driving Immune Exclusion in Hepatocellular Carcinoma**

**Running title: NOTCH3-Mediated Capillary–mCAF Crosstalk Drives Immune Exclusion in HCC**

Fansen Ji^1,2#^, Haochen Li^3#^, Qi Wang^1#^, Jiawei Zhang^4^, Ying Xiao^5^, Huan Li^5^, Hao Liu^5^, Tanqing Long^6^, Boyang Wu^1^, Hao Chen^1^, Haoming Xia^1^, Xinquan Liu^7, 8^, Chuanrui Xu^6^, Yibo Gao^7, 8^, Bingjun Tang^1,*^, Juan Liu^1,*^, Shizhong Yang^1,*^ , Jiahong Dong^1,*^

^1^ Hepatopancreatobiliary Center, Beijing Tsinghua Changgung Hospital, Key Laboratory of Digital Intelligence Hepatology (Ministry of Education), School of Clinical Medicine, Tsinghua Medicine, Tsinghua University, 102218, Beijing, China.

^2^ Tsinghua-Peking Center for Life Sciences. 100084, Beijing, China.

^3^ School of Medicine, Tsinghua Medicine, Tsinghua University, 100084, Beijing, China.

^4^ Health Management Center, Beijing Jishuitan Hospital, Capital Medical University, 100035, Beijing, China.

^5^ Department of Pathology, Beijing Tsinghua Changgung Hospital, Tsinghua University, 102218, Beijing, China.

^6^ School of Pharmacy, Tongji Medical College, Huazhong University of Science and Technology, 430030, Wuhan, Hubei, China.

^7^ Department of Thoracic Surgery, National Cancer Center/National Clinical Research Center for Cancer/Cancer Hospital, Chinese Academy of Medical Sciences and Peking Union Medical College, Beijing, 100021, China.

^8^ Central Laboratory & Shenzhen Key Laboratory of Epigenetics and Precision Medicine for Cancers, National Cancer Center/National Clinical Research Center for Cancer/Cancer Hospital & Shenzhen Hospital, Chinese Academy of Medical Sciences and Peking Union Medical College, 518116, Shenzhen, China.

^#^ These authors contributed equally: Fansen Ji, Haochen Li, Qi Wang

* Correspondence: [dongjiahong@tsinghua.edu.cn](mailto:dongjiahong@tsinghua.edu.cn) (Jiahong Dong), [ysza02008@btch.edu.cn](mailto:ysza02008@btch.edu.cn) (Shizhong Yang), [lja02720@btch.edu.cn](mailto:lja02720@btch.edu.cn) (Juan Liu), [tbja03642@btch.edu.cn](mailto:tbja03642@btch.edu.cn) (Bingjun Tang)

**SUPPORTING INFORMATION**

**Human Subjects and Sample Collection.**

Human FFPE tissue samples from surgically resected primary HCC patients were collected from department of Pathology at Beijing Tsinghua Changgung Hospital retrospectively. Ethical approval was granted by the Ethics Committee of Beijing Tsinghua Changgung Hospital, Tsinghua University (Approval No. 23642-0-01), and written informed consent was obtained from all participants. Malignant foci, invasive boundaries, and adjacent normal parenchyma tissues were identified and collected by board-certified pathologists and manual annotation.

**Pathological ROI and FOV Selection for Spatial Multi-Omics Profiling.**

To improve spatial profiling throughput, 104 tissue cores (each 2 mm in diameter) were assembled into three tissue microarray (TMA) slides for spatial transcriptomics profiling, and an additional 50 cores were arrayed into one separate TMA slide for spatial proteomics profiling. Five-micron section core were cut and mounted on slides (Leica Apex Bond). The tissue cores (also named regions of interest, ROIs in our study) was defined according to standard histopathological criteria and annotated by board-certified pathologists with expertise in liver pathology. ROI selected for the CosMx assay were guided by matched H&E-stained sections from adjacent tissue slices. To account for intra-tumor heterogeneity, tumor cores were preferentially taken from areas exhibiting representative malignant histomorphology, avoiding regions of extensive necrosis, hemorrhage, or poor tissue preservation.

Tumor (T), normal (N), and tumor-normal boundary (B) regions were first delineated on H&E slides through pathological evaluation and subsequently mapped to the corresponding CosMx imaging fields based on spatial correspondence. Tumor regions were defined by the presence of malignant hepatocytic architecture inside the boundary within 500um, normal regions by preserved liver lobular structures outside the boundary within 500um, and boundary regions by transitional histological features between tumor and adjacent non-tumoral tissue. Cells within each region inherited the corresponding regional label. For cells located at the invasive boundary, annotation was determined by integrating histological boundaries by pathologist-defined regions along with CosMx SMI spatial transcriptomics imaging. No more than three tissue cores were selected per patient. Specifically, we overlaid the spatial expression of established HCC-related marker genes, including CTNNB1, MKI67, TP53, and CD34, across each field of view (FOV). These genes are well-recognized as being involved in HCC development and progression [1, 2]. Based on their spatial enrichment patterns, FOVs with uniform enrichment of these markers were classified as tumor regions, those with uniform depletion as normal regions, and those exhibiting mixed enrichment and depletion patterns as boundary regions. This molecular-level validation was fully consistent with the pathologists’ histological annotations. FOVs were carefully selected to avoid tissue folds, necrotic areas, and regions with low cellularity, ensuring adequate representation of Tumor, Boundary, and Normal regions across samples. Quality control filters were applied to exclude FOVs in which less than 50% of the original cellular composition was retained or that contained fewer than 150 cells after filtering.

**CosMx™ Spatial Molecular Imaging (SMI) Profiling.**

CosMx™ Human Universal Cell Characterization RNA Panel (1000-plex, Bruker) was applied to two TMA slides while CosMx™ Human Universal Cell Characterization RNA Panel (6000-plex, Bruker) was applied to 1 TMA slide. After baking at 60°C overnight, each slide was then undergoing deparaffinization and heat-induced epitope retrieval (HIER) using the Leica Bond RXm system, with ER2 epitope retrieval buffer (Tris/EDTA-based, pH 9.0, Leica Biosystems) at 100°C for 15 minutes. This was followed by enzymatic digestion with 3 μg/ml Proteinase K diluted in ACD Protease Plus at 40°C for 30 minutes. Next, tissue sections were washed twice with diethyl pyrocarbonate (DEPC)-treated water and incubated with 0.00075% fiducials (Bangs Laboratory) in 2X saline sodium citrate containing 0.001% Tween-20 (SSCT) for 5 minutes at room temperature in the dark. Excess fiducials were removed by rinsing with 1X PBS, and the sections were then fixed in 10% neutral buffered formalin (NBF) for 5 minutes at room temperature. Following fixation, the sections were washed twice with Tris-glycine buffer (0.1 M glycine, 0.1 M Tris-base in DEPC H2O) and once with 1X PBS for 5 minutes each. To block reactive sites, the sections were treated with 100 mM N-succinimidyl (acetylthio) acetate (NHS-acetate, Thermo Fisher Scientific) in NHS-acetate buffer (0.1 M NaP, 0.1% Tween, pH 8.0, in DEPC H2O) for 15 minutes at room temperature. After rinsing with 2X SSC for 5 minutes, an Adhesive SecureSeal Hybridization Chamber (Grace Bio-Labs) was applied to the tissue for further processing.

NanoString® in situ hybridization (ISH) probes were prepared by heating at 95°C for 2 minutes, followed by immediate cooling on ice. The ISH probe mixture, consisting of 1 nM 1000/6000-plex ISH probe, 10 nM attenuation probes, 1X Buffer R, and 0.1 U/μL SUPERase•In™ (Thermo Fisher Scientific) in DEPC-treated water, was carefully pipetted into the hybridization chamber. To prevent evaporation, the chamber was securely sealed, and hybridization was carried out at 37°C overnight. Following hybridization, tissue sections underwent two washes in 50% formamide (VWR) diluted in 2X SSC at 37°C for 25 minutes. This was followed by two additional washes with 2X SSC for 2 minutes each at room temperature. The sections were then blocked with 100 mM NHS-acetate in the dark for 15 minutes. Finally, a custom-made flow cell was affixed to the slide in preparation for loading onto the CosMx SMI instrument.

**CosMx Spatial Molecular Imager (SMI) Instrument Run**

The assembled flow cell was mounted onto the imaging instrument, and Reporter Wash Buffer was introduced to remove any air bubbles. An initial full-field scan was performed. To initiate RNA readout, 100 μL of Reporter Pool 1 was loaded into the flow cell and incubated for 15 minutes. Unbound reporter probes were removed by washing with 1 mL of Reporter Wash Buffer, followed by the addition of Imaging Buffer to enable fluorescence imaging. For each field of view (FoV), nine Z-stack images were acquired at 0.8 μm intervals. Subsequently, photocleavable linkers on the fluorophore-labeled reporter probes were cleaved via UV illumination, and the flow cell was washed with Strip Wash Buffer. This hybridization and imaging cycle was repeated for all 16 reporter pools in the 1000-plex panel, and for 27 reporter pools in the 6000-plex panel, enabling highly multiplexed RNA detection through iterative rounds of probe hybridization and imaging.

Following RNA readout, tissue samples were incubated for 2 hours within the CosM™SMI instrument for morphological visualization and cell segmentation using a four fluorophore conjugated antibody cocktail targeting CD298/B2M (488 nm), PanCK (532 nm), CD45 (594 nm), and CD3/CD68 (647 nm), along with DAPI staining. After incubation, unbound antibodies and excess DAPI were removed through washes with Reporter Wash Buffer. Imaging Buffer was then introduced into the flow cell, and nine Z-stack images were acquired across five channels corresponding to the four antibodies and DAPI.

**Cell Segmentation**

Image segmentation was based on an enhanced version of the CellPose algorithm [3] by combining information from cell membrane proteins, nuclei and RNA, integrating signals from membrane proteins, nuclear staining, and RNA transcripts to accurately delineate cell boundaries. Following segmentation and transcript assignment at the single-cell level, a transcript-by-cell count matrix was generated. Cells were excluded from downstream analysis if the proportion of negative control transcripts exceeded 1% or if system control counts were greater than 20%.

Specifically, cell segmentation was performed using Nanostring AtoMx Spatial Informatics Platform, which is specifically optimized for CosMx imaging-based spatial transcriptomics data. Briefly, segmentation was based on multi-channel morphological information including nuclear staining (DAPI) and membrane markers included in the CosMx morphology panel, allowing robust delineation of individual cells in densely packed tumor regions. For the initial segmentation, AtoMx applies a nucleus-centered, watershed-based algorithm in which nuclei are first identified and then expanded to approximate whole-cell boundaries using membrane signal guidance. Key parameters, including the expected cell diameter range, were set according to tissue type and histopathological characteristics of liver tumor specimens, following NanoString’s recommended settings for solid tissues. These parameters were kept consistent across all samples to avoid introducing sample-specific bias. Specifically, we applied Configuration A (Non-Neuro Human Tissue) with the following settings: Nucleus Label diameter of 7.2 μm, Cytoplasm Label diameter of 8.28 μm. For Mask Processing, we used a Foreground Threshold of 0, Nearest Neighbor Split set to False, and a minimum/maximum diameter ratio of 0.25 and 4 respectively. Nucleus Label Options with Model bsbNuc, Probability Threshold of 0, and Flow Threshold of 0.4. Cytoplasm Label Options with Model bsbCyto, Probability Threshold of 0, and Flow Threshold of 0.4. Final Cell Label minimum size of 3.5 μm and dilation/erosion of 2.16 μm. Segmentation parameters were applied uniformly across all FOVs. Segmented objects showing incomplete nuclear signals or clear overlap artifacts were excluded during downstream QC.

**Preprocessing, Integration and Dimension Reduction of CosMx SMI data.**

To integrate data from multiple patients, the original dataset was split into individual Seurat objects per patient, retaining both spatial metadata and expression matrices. Integration was performed using the IntegrateLayers function, which identifies anchor features and applies batch correction while preserving spatial context. All UMAP visualizations presented in this study were generated solely based on gene expression profiles, without incorporating any spatial information or spatial priors.

**Quality control and cell filtering.**
Single-cell spatial transcriptomic data were processed using the Seurat package (v5.0.1) [4]. Library size normalization and SCTransform were first applied to each dataset. Cells with percent of system control large than 20% or percent of negative control larger than 1% were filtered out. For CosMx 1K data, cells with low complexity/library size (less than 100 expressed genes or a total sum of counts across all genes below 200) were filtered out. For CosMx 6K data, cells with less than a total of 300 gene counts or unique gene features less than 200 were filtered out.

**Batch-effect correction.**
To mitigate batch effects arising from different patients, gene expression data were normalized using the regularized negative binomial regression model (SCTransform, SCT from Seurat 5.0.1 package) in order to account for technical artifacts while preserving biological variance. Batch correction was performed using the RPCAIntegration method integrated in the IntegrateLayers function with each patient as a batch. Batch correction performance was evaluated by visual inspection of UMAP embeddings colored by batch and by assessing the mixing of cells across batches within major cell types, confirming that biological structure was preserved while technical variation was reduced. The integrated dataset was then scaled across all genes and subjected to dimensionality reduction using PCA, followed by UMAP for visualization. Based on the elbow plot (percentage variance explained vs. number of principal components), the top 30 PCs were selected for downstream analysis. The resulting integrated dataset was used for subsequent clustering, differential expression analysis, and spatial interaction profiling.

**Cell Type Annotation**
Cell type annotation was performed in two steps, combining canonical marker genes with cluster-specific highly expressed genes. First, major cell lineages were identified using known markers:

- T/NK cells: PTPRC, CD3D, CD3E, NKG7, NCAM1, KLRG1, KLRB1
- B/plasma cells: CD79A, CD19, MS4A1, MZB1, IGHA1, JCHAIN, SDC1
- Myeloid cells: CD14, CD16, CD68, CD163
- Endothelial cells: CDH5, VWF, PECAM1
- Fibroblasts: ACTA2, COL1A1, COL1A2, DCN
- Malignant/hepatocytes: TTR, SAA1/2, FGG

In the second step, each lineage was subsetted and re-clustered to identify subpopulations. The FindAllMarkers function in the Seurat package was applied using highly variable genes (HVGs) and filtered for positive log-fold changes. The top 100 differentially expressed genes were used to define cell subtypes. Clusters expressing markers from multiple lineages were excluded as potential multiplets resulting from inaccurate segmentation or ambient RNA contamination.

**Malignant hepatocytes and CAF subtypes annotation.**

We adopted a primary criterion to combine the spatial context and histopathological annotation to accurately define malignant/non-malignant hepatocytes. Specifically, tumor regions and peri-tumoral regions were first delineated based on matched H&E staining and pathological evaluation at the ROI level. Hepatocytes located within pathologist-annotated tumor regions were defined as malignant hepatocytes, whereas hepatocytes within peri-tumoral regions were defined as adjacent non-malignant hepatocytes. For hepatocytes located at the invasive margin region, cell identity was labeled as “Boundary” and was further evaluated by integrating transcriptomic similarity from clustering analysis among all hepatocytes (malignant and non-malignant cells) as shown in Figure 2E. These cells were assigned based on their clustering relationships and nearest-neighbor similarity to either tumor-associated or peri-tumoral hepatocyte populations, thereby minimizing ambiguity at the boundary regions. In order to define invasive boundary in a more concise way, we labeled all transcripts of CTNNB1, MKI67, TP53 and CD34 to each FOV, which were recognized as marker genes involved in HCC development [1, 2] as shown in Figure 1B to validate the pathologists’ annotation. Cells in FOVs where total transcripts of these four genes were enriched were defined as malignant while Cells in FOVs where total transcripts of these four genes were depleted were defined as adjacent normal hepatocytes. Using this method, the invasive front at the FOV image view could be outlined apparently, making it possible for us to define the Boundary at the FOV level. Further cell identity annotation from these FOVs were performed by clustering analysis.

In this study, the classification of CAF subtypes was performed strictly based on well-established marker gene sets reported in multiple independent studies across HCC and other solid tumors. Rather than proposing novel CAF categories, we adopted a literature-driven annotation strategy to ensure biological interpretability and comparability. It is also important to clarify the nature of the tissue samples used for spatial profiling. All specimens were formalin-fixed paraffin-embedded (FFPE) tissues obtained from surgical resections of primary HCC. Although spatial regions adjacent to the tumor were included, these areas should not be considered as truly normal liver tissue, as they are inevitably influenced by the tumor microenvironment, chronic liver disease background, and local inflammatory remodeling. Consistent with this biological context, our cell-type annotation revealed that fibroblasts enriched in the adjacent regions predominantly exhibited an inflammatory CAF like transcriptional profile. Therefore, we did not define or annotate a population of normal hepatic stellate cells, as such a designation would not be biologically appropriate for the samples analyzed.

**High-Plex Spatial Proteomics via GeoMx DSP Protein Assay**

High-plex spatial proteomic profiling was conducted using the GeoMx Digital Spatial Profiler (DSP; Bruker), which combines conventional immunofluorescence imaging with digital optical barcoding to enable spatially resolved, multiplexed protein quantification. Sample preparation for DSP followed standard protocols to ensure optimal antigen retrieval and minimize non-specific binding. FFPE TAM slide containing 50 cores were first deparaffinized in xylene three times with 5-10 min each, rehydrated through graded ethanol (100%, 95%, 70%, each for 5-10 min), and rinsed with distilled water. Antigen retrieval was then performed in citrate buffer (0.01 M, pH 6.0) using a pressure cooker at 95-100 °C for 10-30 minutes to reverse formalin-induced cross-links. Slides were then blocked with Buffer W at room temperature to reduce non-specific antibody binding.

Tissues were subsequently incubated overnight with a cocktail of antibodies conjugated to photocleavable oligonucleotide tags, along with fluorescent morphology markers for α-SMA (647/666 nm), CD45 (594/615 nm), CK8/18 (532/568 nm) and SYTO13 (488/525 nm) for compartment visualization. Slides were imaged at 20× resolution using the GeoMx DSP system to visualize tissue architecture and delineate regions of interest (ROIs). In total, 95 CAF-enriched ROIs were manually annotated per tissue core across malignant foci, invasive boundaries, and adjacent normal parenchyma. Then, the GeoMx Immune-Oncology (IO) Proteome Assay comprising up to 570 immunohistochemistry validated antibodies conjugated to photocleavable oligonucleotide tags was applied to the tissue sections. Following UV exposure, oligonucleotide tags from each ROI were released, collected into a plate, and subjected to PCR amplification using GeoMx Seq Code primers. Amplified libraries were pooled, purified, quality-checked, and perform next-generation sequencing (NGS) using the Illumina NovaSeq platform (Illumina) for high-plex protein quantification. Finally, the sequencing counts were mapped back to their original spatial coordinates, generating high-resolution digital proteomic maps.

**GeoMx DSP data Processing and Analysis**

Raw sequencing data were first processed by trimming adapters, merging reads, and aligning sequences to identify target probes. Unique molecular identifiers (UMIs) were used to eliminate PCR duplicates, generating accurate digital counts. For each sample, protein-level expression was calculated by aggregating probe signals and excluding outliers, with the final value defined as the median signal after removing outlier probe. The limit of quantification was set as the log-mean of negative control probes plus two standard deviations. Inter-sample variation was assessed using L2 norm distance. Antibody specificity was evaluated by calculating the signal-to-noise ratio (SNR), defined as the target signal divided by the mean of triplicate IgG controls in matched regions; proteins with SNR < 3 were considered undetectable. Fold changes and differential gene expression were reported for each protein using t.test() function in R.

**Quantification of Cell Type Proportions Using HALO Software**

Morphology markers-stained GeoMx tissue slide was scanned at high resolution and analyzed using the HALO^®^ image analysis platform (Indica Labs). A dedicated multiplex analysis module was employed to segment individual cells based on DAPI nuclear staining and to identify cell types using specific fluorescence markers: CK8/18 for epithelial cells, α-SMA for stromal cells, and CD45 for immune cells. Signal thresholds for each marker were manually optimized and validated by a board-certified pathologist to ensure accurate cell classification. The relative proportion of each cell type was calculated as the number of positive cells for a given marker divided by the total number of nucleated cells within each ROI. Data were exported from HALO for downstream statistical analysis.

**Colocalization Analysis**

The 𝐶𝐿𝑄_𝑏→𝑎_ is a quantitative metric used to measure the spatial co-occurrence of two cell types: the "target" cell type (denoted as cell type ***a***) and the "infiltrating" cell type (denoted as cell type ***b***). This metric captures the extent to which cell type ***b*** co-localizes with cell type ***a*** within a neighborhood of 50 cells. Specifically, the 𝐶𝐿𝑄 is calculated as the ratio of the observed number of cell type ***b*** cells within a neighborhood of 50 cells of cell type ***a*** to the expected number of cell type ***b*** cells, based on random distribution [5].

Mathematically, the 𝐶𝐿𝑄 is defined as:

$${CLQ}_{b\to a}= \frac{C_{b\to a}/N_{a}}{N_{b}/(N-1)}$$

Where:

- 𝐶_b→a_ represents the number of cells of type ***b*** within a neighborhood of 50 cells of type ***a*.**
- 𝑁 is the total number of cells in the tissue sample.
- 𝑁_a_ and 𝑁_b_ are the total numbers of cells of types a and b, respectively.

It is important to note that 𝐶𝐿𝑄_b→a_ ≠ 𝐶𝐿𝑄_a→b_, as the spatial co-occurrence is not symmetric.

**Pan-cancer Integration of CAFs from scRNA-seq Datasets under ICB Treatment.**

Single-cell RNA-seq datasets related to immune checkpoint blockade (ICB) therapy were retrieved from scICB database [6]. CAFs were extracted from tumor samples and merged across studies. Gene expression was normalized per cell, and the top 3,000 highly variable genes were selected for scaling using Seurat (v5.0.3). PCA was performed, and the top 30 PCs were used for dataset integration via the IntegrateLayers function with reciprocal PCA (RPCA) to correct for batch effects. Dimensionality reduction was carried out using UMAP based on the integrated RPCA space. Clustering was conducted using shared nearest neighbor (SNN) graph-based methods (FindNeighbors and FindClusters). Differentially expressed genes (DEGs) for each cluster were identified using the FindAllMarkers function (Wilcoxon rank-sum test), selecting genes expressed in ≥ 25% of cells with log2 fold change ≥ 0.5 and adjusted *p* <0.05. Cell type annotation was based on canonical marker genes reported in the literature. The CAF subtypes for both scRNA-seq and spatial transcriptomics data included: mCAFs (*COL6A3*, *FN1, MMP14, MMP11*), iCAFs (*DPT*, *CXCL9*, *CXCL10*, *CXCL11*, *CXCL12*, *CCL21*, *EFEMP1*, *PDPN*, *PDGFRA*), aCAFs (*CD74*, *HLA-DRB1*, *HLA-DRA*), vCAFs (*MYH11, DSTN, MUSTN1, ADIRF*), pCAFs (*STMN1, TOP2A, MKI67, CCNB1, TYMS*) and pericytes (*ACTA2, PDGFRB, CSPG4, RGS5*).

**DLL4 Stimulation of LX-2 Cells**

Recombinant human DLL4-Fc (Novoprotein Scientific) was immobilized onto 12-well tissue culture plates by passive adsorption. DLL4-Fc was diluted in sterile PBS to a final concentration of 10 μg/mL, and 500 μL was added to each well, followed by overnight incubation at 4 °C. Control wells received PBS only. The next day, wells were washed twice with PBS to remove unbound proteins and blocked with 1% BSA in PBS for 1 h at room temperature, then rinsed once with PBS. LX-2 cells (Cat# GCL-0560, RRID: CVCL_5792) were seeded at a density of 5 × 10⁵/well and cultured in DMEM supplemented with 10% FBS for 48 h. Cells were harvested for RNA extraction, reverse transcription, and quantitative PCR analysis of NOTCH downstream target (*HES1*) and collagen related genes (*FN1, NOTCH3 and COL4A2*). In parallel, protein lysates were prepared using RIPA buffer supplemented with protease inhibitors, and Western blotting was performed to assess the protein levels. All experiments were performed in at least three biological replicates. Data are presented as mean ± SD, and statistical significance was determined using two-tailed Student’s t-test (*p* < 0.05).

**Construction and Infection of *NOTCH3* shRNA Lentivirus in LX-2 Cells**

To knock down *NOTCH3* expression, short hairpin RNA (shRNA) sequences targeting human NOTCH3 were designed and cloned into a lentiviral vector (pLKO.1) under the control of the U6 promoter. A non-targeting scrambled shRNA sequence was used as a negative control. Lentiviral particles were produced by co-transfecting HEK293T cells with the shRNA plasmid, psPAX2 (packaging plasmid), and pMD2.G (envelope plasmid) using a standard calcium phosphate or Lipofectamine 2000 transfection method. After 48-72 hours, viral supernatants were collected, filtered (0.45 µm), and concentrated if necessary. LX-2 cells were seeded and infected with lentiviral supernatants in the presence of 8 µg/mL polybrene. After 24-48 hours, infected cells were selected using puromycin (1-2 µg/mL) for 3-5 days to establish stable knockdown lines. Knockdown efficiency was confirmed by quantitative RT-PCR and Western blot analysis. NOTCH3-specific short hairpin RNA (shRNA) sequences and scrambled shRNA were synthesized by Sangon Biotech (Shanghai, China). The *shRNA* sequences were as follows:

*shRNA1:* 5′-CGCTGCCTGGACAAGATCAAT-3′;

*shRNA2*: 5′- TCTGGACAAGATCGATGGCTA-3′;

*shRNA3*: 5′- CCGGGACATCACGGATCATA-3′; and

scramble control shRNA: 5′- TTCTCCGAACGTGTCACGT-3′.

The PCR primer sequences were as follows:

NOTCH3 forward: 5′-TCTGGACAAGATCGATGGCTA-3′

NOTCH3 reverse: 5′- CCGGGACATCACGGATCATAT-3′

β-actin forward: 5′-CACCATTGGCAATGAGCGGTTC-3′

β-actin reverse: 5′-AGGTCTTTGCGGATGTCCACGT-3′

**Generation of *DLL4*-overexpressing SK-Hep1 cell line**

To generate a stable *DLL4*-overexpressing liver sinusoidal endothelial cell line, SK-Hep1 cells were transduced with a lentiviral vector encoding the full-length human DLL4 cDNA (pLV-DLL4) or an empty vector control (pLV-Vector) from Sangon Biotech (Shanghai, China). Lentiviruses were produced by co-transfecting HEK293T packaging cells with the plasmid of interest and packaging plasmids using Lipofectamine 3000 (Thermo Fisher Scientific), following the manufacturer’s protocol. Viral supernatants were collected 48 and 72 hours post-transfection, filtered through a 0.45 μm membrane, and used to infect SK-Hep1 cells in the presence of 8 μg/mL polybrene. Following infection, cells were selected with puromycin (2 μg/mL) for 7-10 days to obtain stable clones. Overexpression of *DLL4* was confirmed by quantitative real-time PCR (Table S14) and Western blotting. Control cells were established in parallel using the same protocol with the empty vector.

**LX-2 and SK-Hep1 Co-culture Assay**

LX-2 cells were seeded in 6-well plates, and SK-Hep1 cells were seeded in 0.4 μm transwell inserts for indirect co-culture. After 48 hours, LX-2 cells were collected for RNA extraction, and the expression of collagen-related genes was measured by qRT-PCR. To assess the role of DLL4-NOTCH3 signaling in collagen production, LX-2 and SK-Hep1 cells were co-cultured under four conditions:

(1) WT *LX-2* + WT *SK-Hep1*;

(2) NOTCH3 KO *LX-2* + WT *SK-Hep1*;

(3) WT *LX-2* + DLL4-overexpressing *SK-Hep1*;

(4) NOTCH3 KO *LX-2* + DLL4-overexpressing *SK-Hep1*.

**RNA-seq Analysis of NOTCH3-Knockout and Wild-Type LX-2 Cells**

Total RNA was extracted from NOTCH3-knockout and wild-type LX-2 cells using a standard TRIzol protocol (Thermo Fisher, #15596018). Polyadenylated mRNA was then enriched using oligo(dT)-conjugated magnetic beads. The purified mRNA was subsequently fragmented into short pieces by incubation with fragmentation buffer at elevated temperature. First-strand cDNA synthesis was performed using random hexamer primers and reverse transcriptase, followed by second-strand synthesis to generate double-stranded cDNA. After end repair, A-tailing, and adaptor ligation, the cDNA libraries were amplified by PCR and purified (Table S14). RNA integrity was assessed with the Agilent 2100 Bioanalyzer, and only samples with an RNA integrity number (RIN) > 8 were used for sequencing. RNA-seq libraries sequenced on an Illumina NovaSeq 6000 platform to generate paired-end 150 bp reads. Raw sequencing reads were trimmed and filtered using Trimmomatic, and aligned to the human reference genome (GRCh38) using STAR aligner. Gene expression quantification was performed with featureCounts, and differential expression analysis between NOTCH3-KO and WT groups was carried out using DESeq2. Genes with an adjusted *p* < 0.05 and |log₂ fold change| ≥ 1 were considered significantly differentially expressed.

**Western Blot Analysis**
Total cytoplasmic and membrane proteins from cells were extracted using RIPA buffer or a membrane/cytosol protein extraction kit (Beyotime) with protease/phosphatase inhibitors. Protein concentration was measured by BCA assay. Equal amounts of protein were separated by 8−12% SDS-PAGE, transferred to PVDF membranes, and blocked with 5% non-fat milk. Membranes were incubated with primary antibody (1:1000) overnight at 4 °C, followed by HRP-conjugated secondary antibody. Bands were visualized using the ChemiDoc Imaging System (Bio-Rad). Antibodies: NOTCH3 (Cat#ab23426, RRID: AB_776841), FN1 (Cat# ab268020, RRID:AB_2941028), COL4A2 (RRID:AB_10976330), Cd8 (Cat#ab209775, RRID:AB_2860566), α-SMA (Cat#ab124964, RRID:AB_11129103), CD68(Cat#ab283654, RRID:AB_2922954), CD34 (Cat#ab8158, RRID:AB_306316)

**Transwell Co-culture Assay of LX-2 and CD8^+^ T cells**

Human peripheral blood mononuclear cells (PBMCs) were isolated and CD45RO⁺CD8⁺ T cells were sorted by fluorescence-activated cell sorting (FACS). Sorted cells were stimulated with anti-CD3/CD28 beads (1:1) and 100 U/mL IL-2 for 24 hours. After activation, 1 × 10⁵ CD8⁺ T cells were seeded into the upper chamber of a 5 μm pore size transwell insert. The lower chamber was filled with conditioned medium collected from wild-type or NOTCH3^-/-^ LX-2 cells cultured for 24 hours. After a 24-hour co-culture, the number of CD8⁺ T cells that had migrated to the lower chamber was determined by manual cell counting using a hemocytometer.

**ELISA Assay**

Supernatants were collected from the lower chamber of the CD8⁺ T cell transwell co-culture system after 48 hours. The levels of Granzyme B (GZMB), Tumor Necrosis Factor alpha (TNF-α), and Interferon gamma (IFN-γ) were measured using commercially available human ELISA kits (GZMB: E-EL-H1617, TNF-α: E-EL-H0109, IFN-γ: E-EL-H0108, Elabscience), according to the manufacturer’s protocols. Absorbance at 450 nm was measured using a microplate reader, and cytokine concentrations were calculated based on standard curves generated in parallel.

**Orthotopic HCC Implantation Mouse Model**

BALB/c mice (RRID: MGI:2161072) were purchased from the Vital River Laboratory Animal Technology Co. (Beijing) and were 6 weeks old. All procedures involving mice and experimental protocols were approved by Institutional Animal Care and Use Committee Beijing Tsinghua Changguang Hospital (25098-0-01). For the orthotopic HCC implantation model, H22 murine hepatocellular carcinoma cells (5 × 10⁶ cells in 100 μL PBS) were injected subcutaneously into the right flank of 6-8-week-old male BALB/c mice. After tumor establishment, obtained subcutaneous tumors were then sectioned into tissue blocks measuring 2 mm × 2 mm × 2 mm and implanted into the left lobe of the liver in BALB/c mice. Following implantation after 10 days, mice were randomized into four different treatment groups of Vehicle, DAPT (i.p., 10mg/kg, daily), Anti-PD1 (i.p., 10mg/kg, q3d) and DAPT combined with Anti-PD1. After 14 days of treatment, mice were euthanized for tumor harvesting, weighing, and further downstream analyses.

**Endogenously Arising Mouse Tumor Model**

In the C57BL/6 mice (Vital River Laboratory Animal Technology Co., RRID: MGI:2159769) orthotopic tumor model, hydrodynamic tail vein injection was employed to deliver the plasmid complex system. The system was formulated in physiological saline as the vehicle, with final concentrations of c-Akt-pT3EF1αH (7.5 μg/mL), c-myc-pT3EF1αH (7.5 μg/mL), and the Sleeping Beauty (SB) transposon system (pCMV-SB plasmid, 0.6 μg/mL). Administration was performed via tail vein injection at a dose of 0.1 mL/g of body weight. Using a 20 g mouse as an example, inject 2 mL of sterile saline containing 15 µg c-Akt-pT3EF1αH, 15 µg c-myc-pT3EF1αH, and 1.2 µg pCMV-SB via the tail vein within 5–7 s. All these plasmids were kindly provided by Dr. Xin Chen of University of Hawaii Cancer Center. Once tumors were established, mice were randomized into 4 different treatment groups and administered either Vehicle, DAPT (10 mg/kg, intraperitoneally, once daily), anti-PD-1 antibody (10 mg/kg, intraperitoneally, every three days) or DAPT combined with anti-PD-1. After 14 days of treatment, mice were euthanized for tumor harvesting, weighing, and further downstream analyses.

**Flow cytometry analysis**

After treatment, the mice were sacrificed, and their liver tumors were harvested. Tumor tissues were mechanically dissociated and enzymatically digested. The resulting cell suspensions were filtered through a 70 μm filter in RPMI medium and then centrifuged at 400 g for 5 min at 4 °C. The supernatant was discarded, and the remaining cells were incubated at 4 °C for 30 min in ice-cold FACS buffer (1% BSA and 0.5 mM EDTA) with Fc Block diluted 1:30. After washing in FACS buffer, the cells were stained with conjugated antibodies for 30 min at 4°C in the dark to identify various immune cell populations. Intracellular Fixation & Permeabilization Buffer Set (Invitrogen, Cat# 88-8824-00) was utilized to Intracellular staining. The cells were then washed twice more in PBS and resuspended in 450μL of PBS for analysis. The analysis was performed using BD FACSLyric™ Flow Cytometer.

**Cytokine Antibody Array**

To assess plasma cytokine expression profiles of mouse model, we employed the RayBio® Mouse Cytokine Antibody Array (QAM-TH17-1-1, RayBiotech, Norcross, GA, USA) according to the manufacturer’s instructions. This array enables the simultaneous detection of 18 cytokines, including IL-1β, IL-2, IL-4, IL-5, IL-6, IL-10, IL-12p70, IL-13, IL-17, IL-17F, IL-21, IL-22, IL-23, IL-28, IFN-γ, MIP-3α, TGF-β1, and TNF-α. Serum and tissue samples were analyzed in triplicate for each experimental condition. Fluorescence signals were acquired using an Axon GenePix 4000B microarray scanner, and data were quantified with GenePix Pro software.

**mIHC staining of FFPE Mouse HCC Samples**

For FFPE mouse HCC, bake poly-L-lysine-coated slides at 68 °C to melt paraffin, deparaffinize in xylene and rehydrate through graded ethanol to water, then perform heat-induced antigen retrieval in citrate buffer (pH 6.0; pressure cooker or microwave) and cool; wash in PBS. Quench endogenous peroxidase with 3% H₂O₂ (∼15-60 min), wash, block with goat serum (∼20−60 min), then incubate with the first primary antibody (validated in monoplex) overnight at 4 °C. After PBS washes, apply an HRP-conjugated secondary and develop with a tyramide working solution (e.g., Opal dyes) for 5−10 min; stop the reaction with PBS, then perform an antibody-stripping step via brief antigen retrieval to remove bound Igs while retaining the covalently deposited fluorophore. Repeat the primary-HRP-TSA-strip cycle sequentially for the remaining targets to build a five-marker, six-color panel (five Opal fluorophores plus DAPI), assigning brighter fluorophores to low-abundance antigens and spacing emission spectra to minimize bleed-through. After the final cycle, counterstain with DAPI (∼10 min), rinse, mount in antifade, and image with a multispectral system followed by spectral unmixing. Parameters above (bake/dewax/rehydration, citrate retrieval, H₂O₂ quench, serum block, overnight primary, 30 min secondary, 5−10 min fluorophore development, cycling to completion, and DAPI + antifade mount) follow our lab’s standard workflow and are consistent with published TSA-mIHC guidance for 6-plex FFPE staining

**TCGA data analysis**

Survival and gene expression analysis between tumor and normal tissues were conducted using Gepia2 web server [7]. To identify genes positively correlated with *NOTCH3* and *DLL4*, transcriptomic and phenotypic data from TCGA were obtained via the UCSC Xena platform [8]. Pearson correlation coefficients were calculated between the expression levels of *NOTCH3*/*DLL4* and all other genes. Genes with a correlation coefficient (r) > 0.4 and a *p* value < 0.05 were considered significantly positively correlated and selected for subsequent GO enrichment analysis.

**
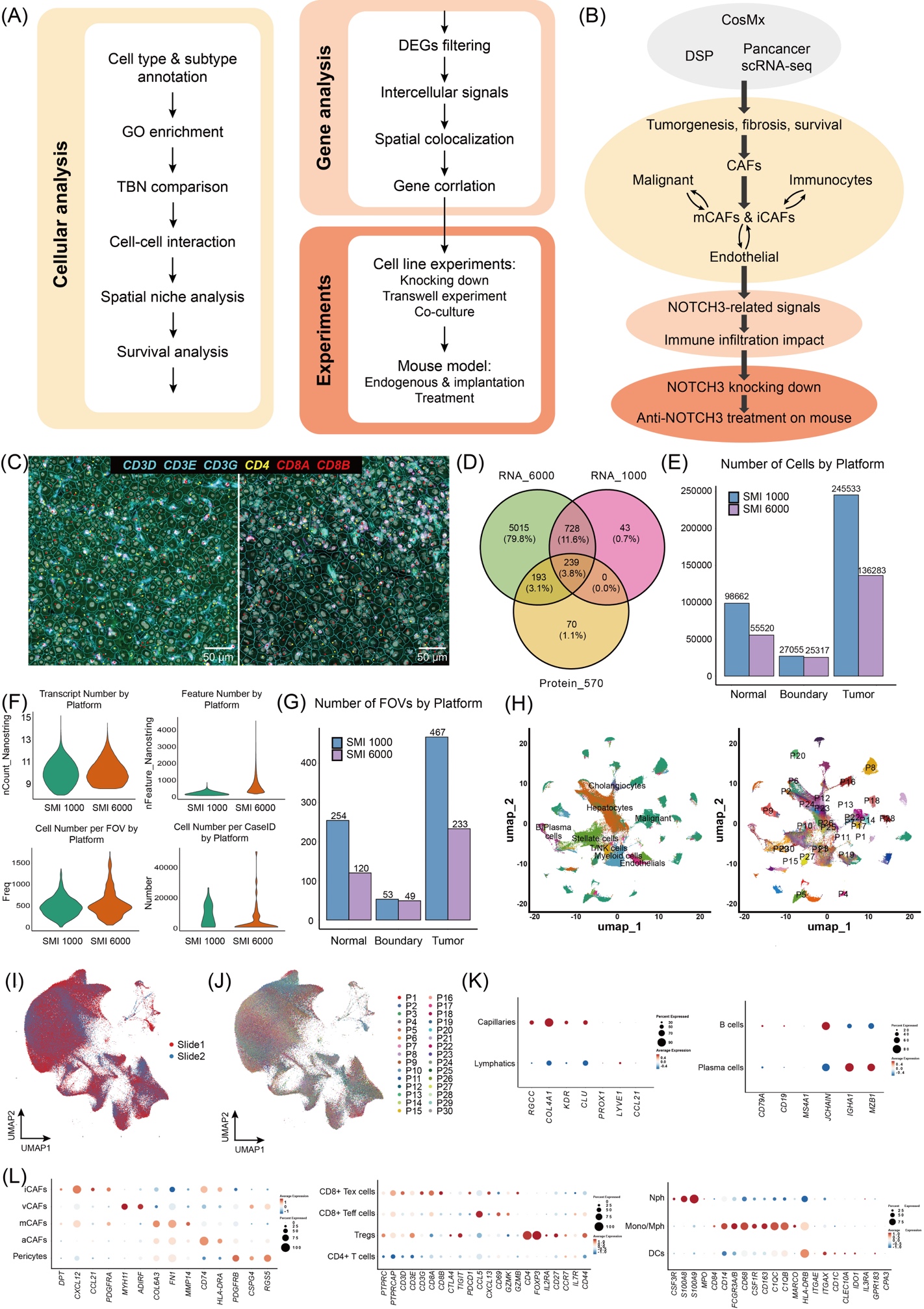
**

**Figure S1 Overview of spatial transcriptomic and proteomic profiling in HCC.**

(A) Summarized pipeline for bioinformatic analysis and experiments of this study. (B) The process of drawing conclusions from data, analysis, and validation**. (C)** CosMx immunofluorescence images showing actual multi-channel overlays, including DAPI, PanCK, CD298, and CD45. (D) The detected genes in CosMx1000, CosMx6000, and GeoMx 570 spatial proteomics datasets. (E) Total number of cells captured per platform (CosMx1000 and CosMx6000)**.** (F) Comparison of transcript number, feature number, cell number per FOV and cell number per CaseID between CosMx1000 and CosMx6000. (G) Number of fields of view (FOVs) captured per platform (CosMx1000 and CosMx6000). (H) UMAP visualization for CosMx1000 data before integration. **(I)** UMAP visualization for CosMx1000 data containing slide1 and slide2. (J) UMAP visualization for patient information in CosMx1000. (K-L) Dot plots showing the marker gene expression of different cell subtypes in CosMx 1000 data.


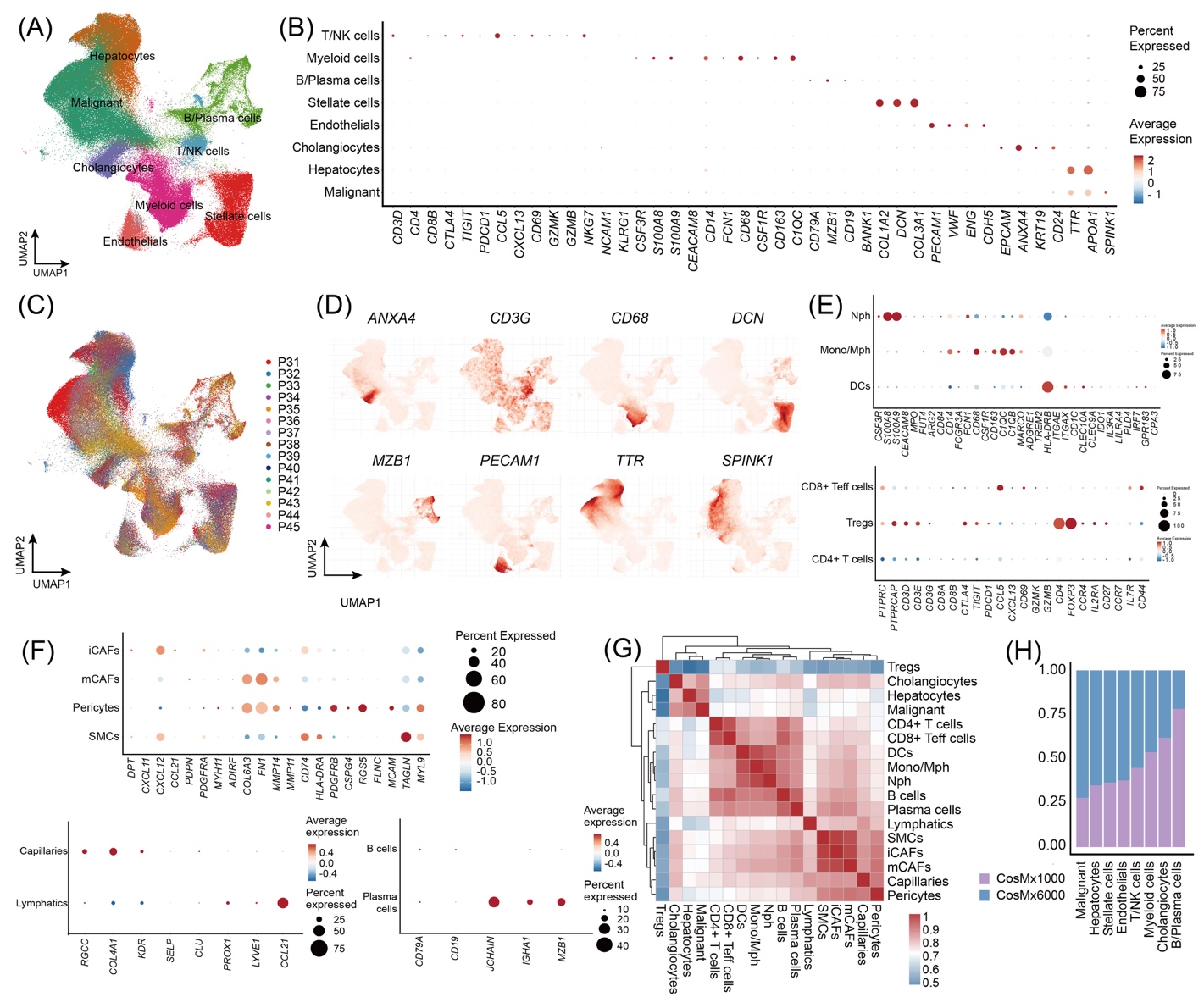


**Figure S2 Overview of CosMx 6000 spatial transcriptomic data.**

(A) UMAP visualization for cell type information in CosMx6000. (B) Dot plot showing the expression of canonical marker genes across CosMx6000 major cell types. (C) UMAP visualization for patient information in CosMx6000. (D) Gene expression profiles of canonical marker genes across major cell types in CosMx6000 data. (E-F) Dot plots showing the marker gene expression of cell subtypes in CosMx 6000 data. (G) Gene expression correlation matrix between cell types in CosMx6000 data**.** (H) The proportion of each cell type derived from CosMx 1000 and 6000.

**
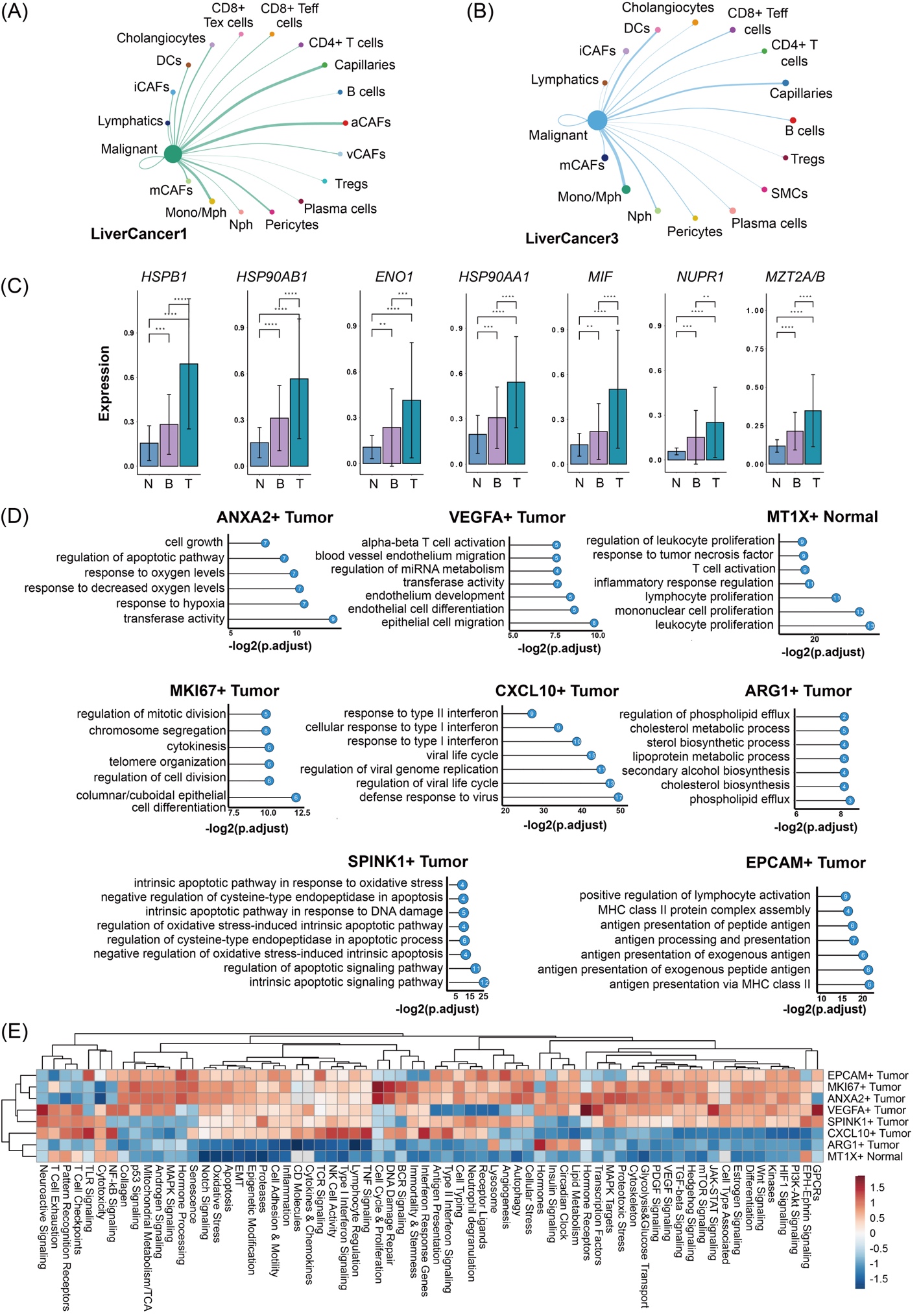
**

**Figure S3 Additional analysis on spatial heterogeneity and interactions of tumor cells.**

(A) Cell-cell communication network analysis of malignant cells and other cell types in Cosmx1000 slide 1. (B) Cell-cell communication network analysis of malignant cells and other cell types in Cosmx6000. (C) Expression levels of key oncogenic and stress response genes across tissue regions (N: normal, B: boundary, T: tumor) in CosMx6000 data. (D) Gene Ontology (GO) enrichment analysis on differentially expressed genes of each hepatocyte subtypes in CosMx1000 data. (E) Heatmap showing the enrichment of key functional gene sets across malignant and normal hepatocyte types. For statistical significance, ^*^*p*  <  0.05, ^**^*p*  <  0.01, ^***^*p*  <  0.001, and ^****^*p* < 0.0001, ns = not significant.


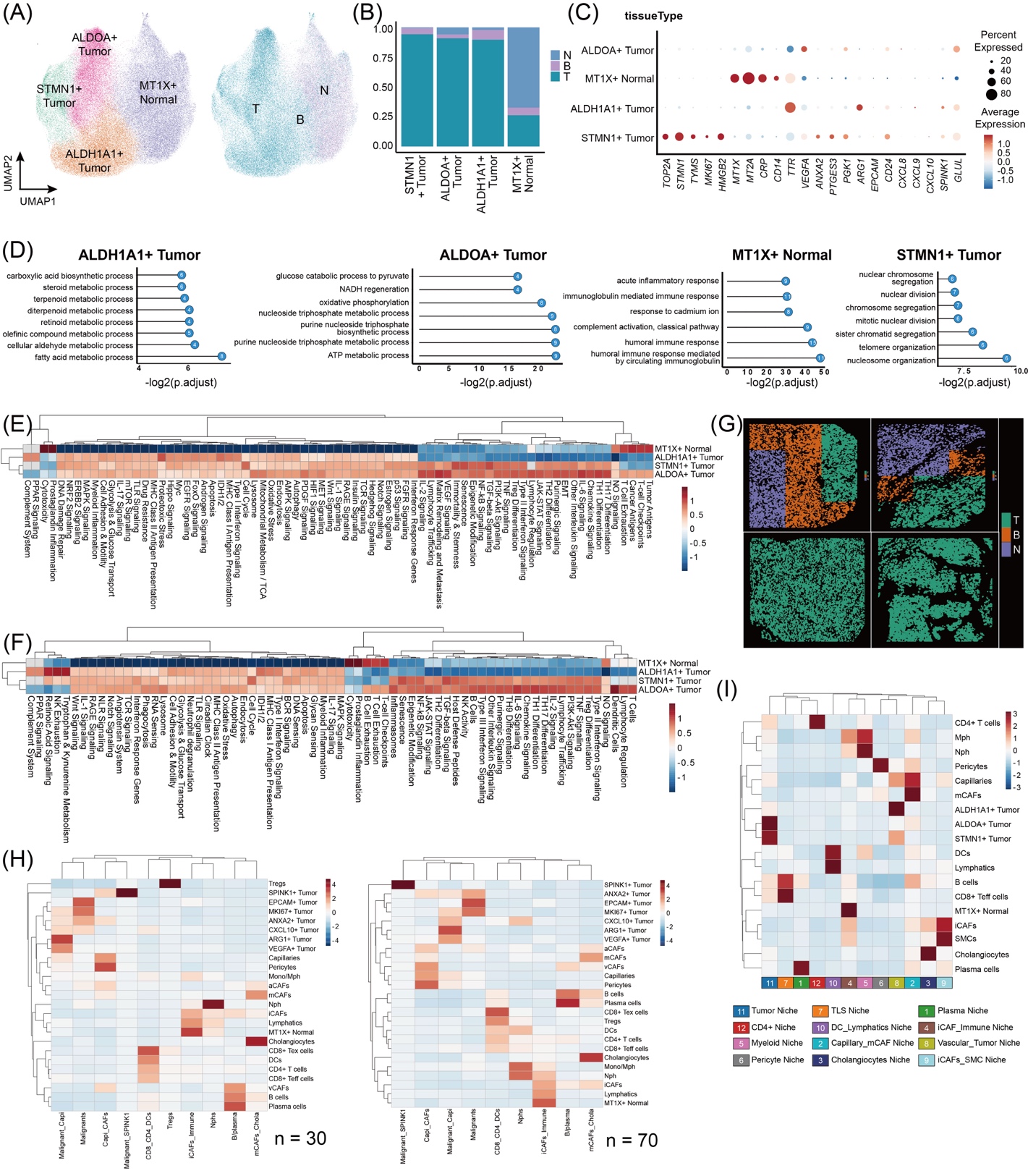


**Figure S4 Analysis on spatial heterogeneity and interactions of tumor cells in CosMx6000 data.**

(A) UMAP visualization of subtypes from all malignant and normal hepatocyte subtypes in Cosmx6000 data. (B) Hepatocyte subtype component of normal, boundary, and tumor regions in Cosmx6000. (C) Dot plot showing the expression of canonical marker genes across hepatocyte subtypes in Cosmx6000. (D) Gene Ontology (GO) enrichment analysis on differentially expressed genes of each hepatocyte subtypes in CosMx 6000 data. (E) Gene set enrichment analysis (GSEA) of tumor-related pathways enriched in different hepatocyte subtypes. (F) GSEA of immune-related biological pathways enriched in different hepatocyte subtypes. (G) Corresponding Tumor (T), Boundary (B), and Normal (N) representation plot of spatial niche in Figure 2H. (H) Spatial niche enrichment using n = 30 and 70 nearest neighbors. (I) Normalized cell type components of identified spatial niches in Cosmx6000.

**
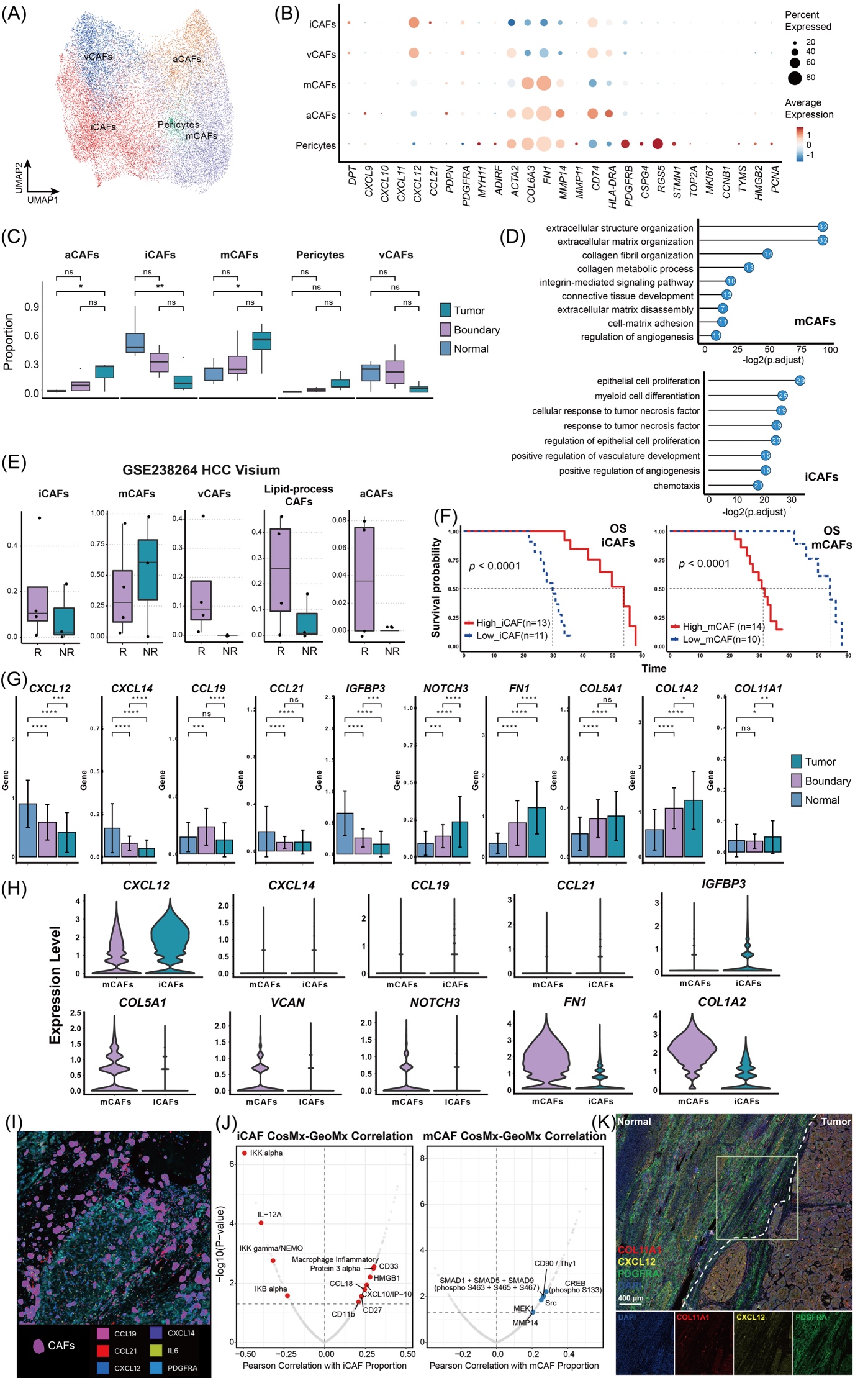
**

**Figure S5 Additional analysis of CAFs polarization and spatial preference in HCC.**

(A) UMAP plot showing the clustering of different CAF subtypes in Cosmx6000. (B) Dot plot representing the average expression levels of select marker genes for each CAF subtype in Cosmx6000 data. (C) Box plots showing the proportion of each CAF subtype across normal (N), boundary (B), and tumoral (T) regions in Cosmx6000 data. (D) The enrichment of Gene Ontology (GO) terms in iCAFs and mCAFs of Cosmx6000 data. (E) Box plots comparing the proportion of each CAF subtype across non-responders (NR) and responders (R) after neoadjuvant cabozantinib and nivolumab in GSE238264 HCC Visium data. (F) Kaplan-Meier survival curves showing the prognostic significance of mCAF and iCAF proportion in an independent validation cohort. (G) Bar plots showing the expression levels of select genes across non-tumoral (N), boundary (B), and tumoral (T) regions in Cosmx6000 data. Statistical significance is indicated by ns (not significant), *p* < 0.05, *p* < 0.01, and *p* < 0.001. (H) Violin plots showing the expression levels of select genes in mCAFs and iCAFs of Cosmx6000 data. (I) Expression of CCL19 and CCL21 with other established iCAF markers (IL6, PDGFRA, CXCL12, and CXCL14) in CosMx data at single-cell resolution. (J) Spatial multi-omics correlation analysis integrating the ROI-level proteomic data (GeoMx DSP) with the inferred spatial abundance of iCAF and mCAF subpopulations derived from our CosMx spatial transcriptomics data. (K) mIHC staining of iCAFs/mCAFs markers demonstrate a preferential enrichment of iCAFs in the peritumoral regions, whereas mCAFs are predominantly enriched within the tumor core. For statistical significance, ^*^*p*  <  0.05, ^**^*p*  <  0.01, ^***^*p*  <  0.001, and ^****^*p*  < 0.0001, ns = not significant.


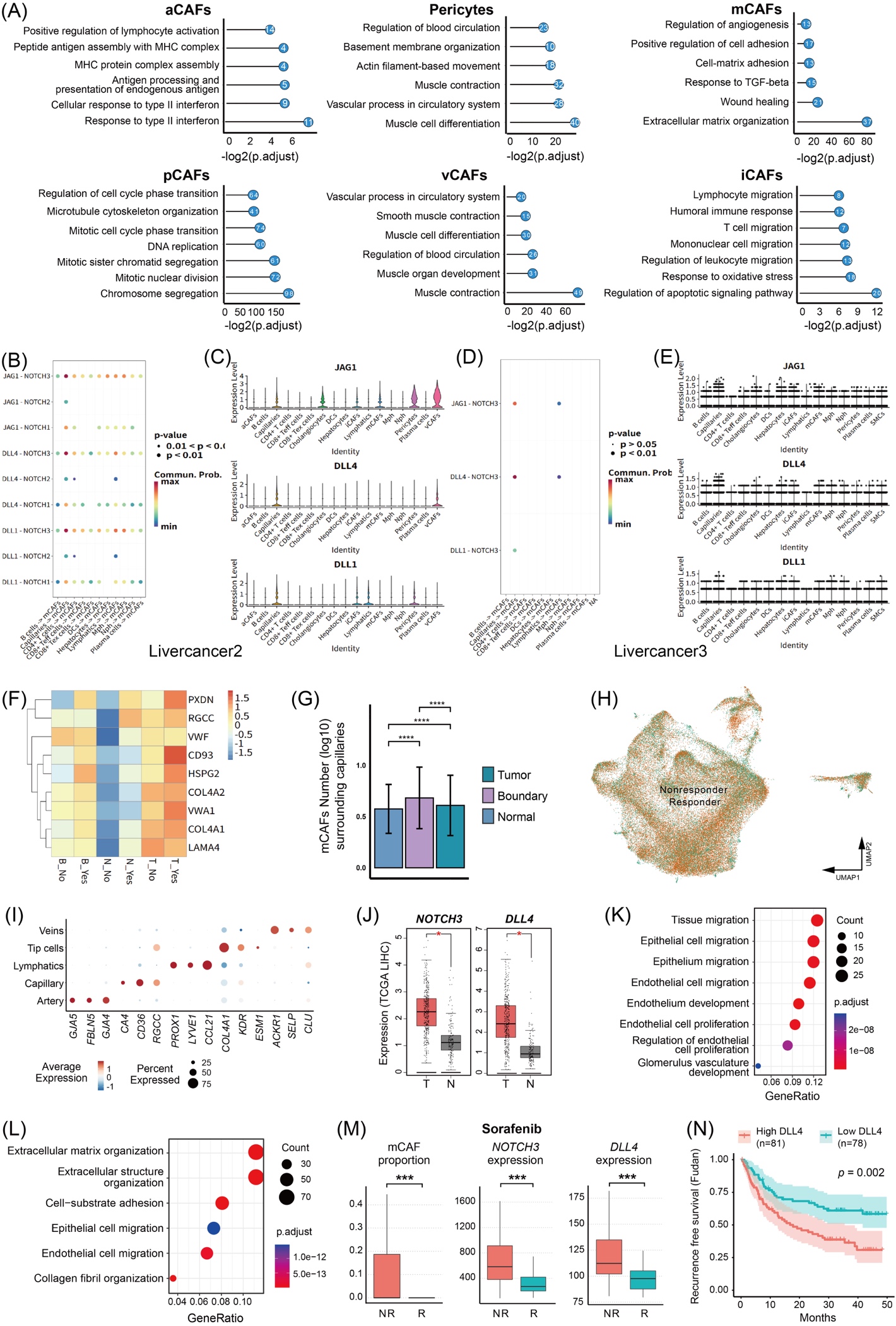


**Figure S6 Detailed analysis of LSECs-mCAFs crosstalk by DLL4/NOTCH3 axis.**

(A) Gene Ontology (GO) enrichment analysis revealing functional divergence among different CAF subtypes by pan-cancer CAFs integration under ICB treatment. (B) CellChat ligand-receptor interaction analysis on NOTCH signaling pathway between capillaries and mCAFs in Cosmx1000 slide 2. (C) Expression levels of DLL1, DLL4, and JAG1 across different cell types in Cosmx1000 slide 2. (D) CellChat ligand-receptor interaction analysis on NOTCH signaling pathway between capillaries and mCAFs in Cosmx6000. (E) Expression levels of DLL1, DLL4, and JAG1 across different cell types in Cosmx6000. (F) Heatmap showing the expression levels of angiogenesis-related genes in LSECs with (Yes) or without (No) spatial proximity of mCAFs in Cosmx6000. (G) mCAF counts in 50 cells surrounding capillaries (log10). (H) UMAP visualization of pan-cancer endothelial cells in different response groups**.** (I) Dot plot visualizing averaged expression of canonical markers across subtypes of endothelial cells by pan-cancer endothelial cell integration under ICB treatment. (J) Comparing the expression levels of *NOTCH3* and *DLL4* between tumor (T) and normal (N) samples in TCGA data. (K, L) Gene ontology (GO) enrichment analysis of genes with significant positive correlation with NOTCH3 and DLL4 in TCGA liver cancer data. (M) Comparing mCAF proportion and the expression levels of *NOTCH3* and *DLL4* between non-responders (NR) and responders (R) to Sorafenib (GSE109211). The proportion of mCAFs in each sample is derived from CIBERSORTx and the deconvolution used our Cosmx1000 data as reference. (N) Kaplan-Meier curves of high- versus low-DLL4 groups for progression-free survival in Fudan liver cancer dataset (OED00094078). For statistical significance, ^*^ *p* < 0.05, ^**^ *p* < 0.01, ^***^ *p* < 0.001, and ^****^ *p* < 0.0001, ns = not significant.


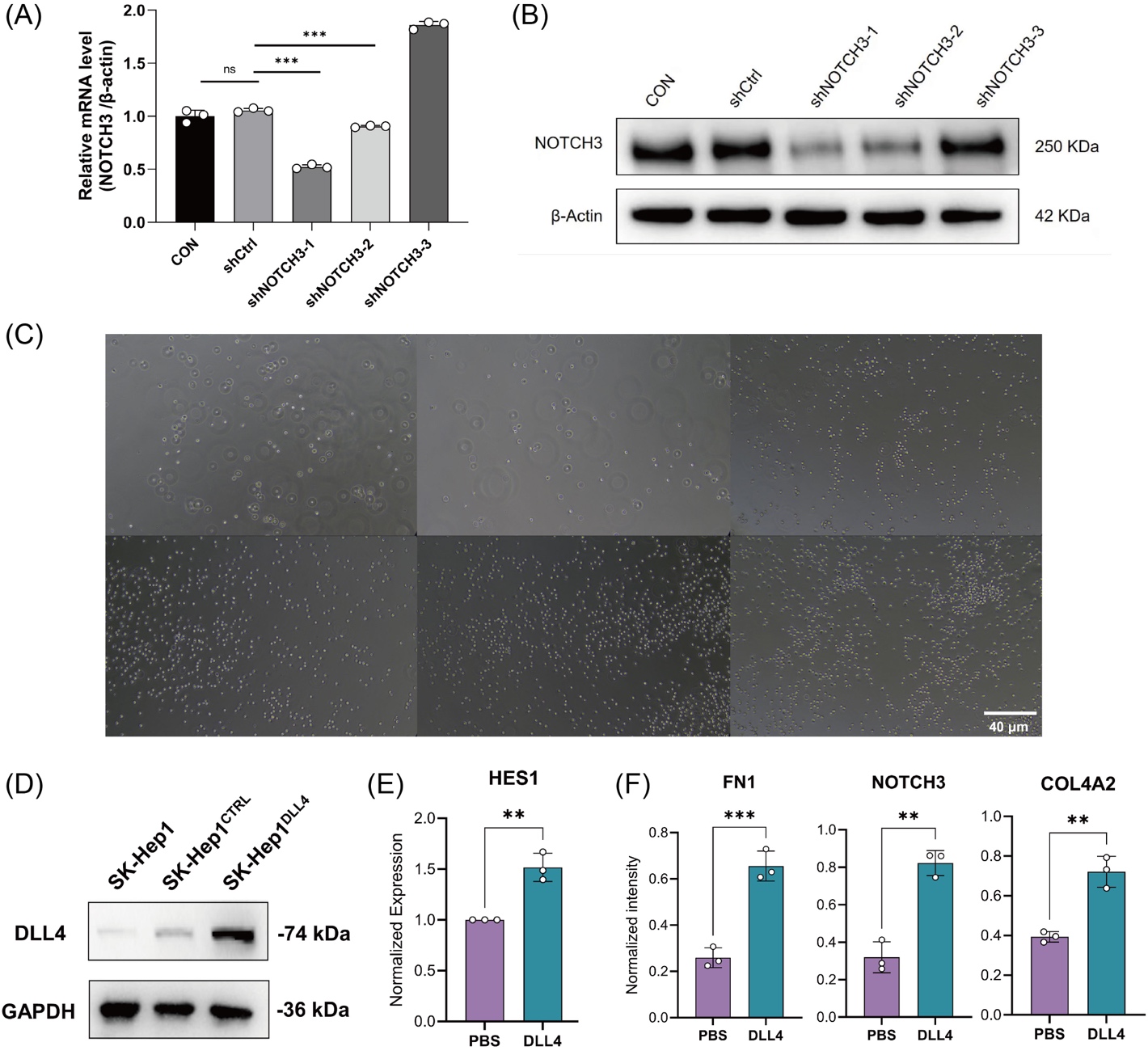


**Figure S7 Experiment validation of LSECs-mCAFs crosstalk by DLL4/NOTCH3 axis.**

(A) Bar plots showing the relative mRNA levels of NOTCH3 in the NOTCH3 knocking down experiment. (B) Western blot analysis showing the levels of NOTCH3 protein in the NOTCH3 knockdown experiment. (C) The number of CD8^+^ cells in the lower chamber before (upper row) and after (lower row) migration of the Transwell experiment. The three columns are blank culture medium, supernatant of LX-2 culture, and supernatant of Notch3-KD LX-2. (D) Western blot analysis showing protein levels in the DLL4-overexpressed SK-Hep1 cell line. (E, F) Comparing HES expression levels and the protein levels of FN1, NOTCH3, and COL4A2 between LX-2 cell line with and without DLL4 stimulation.


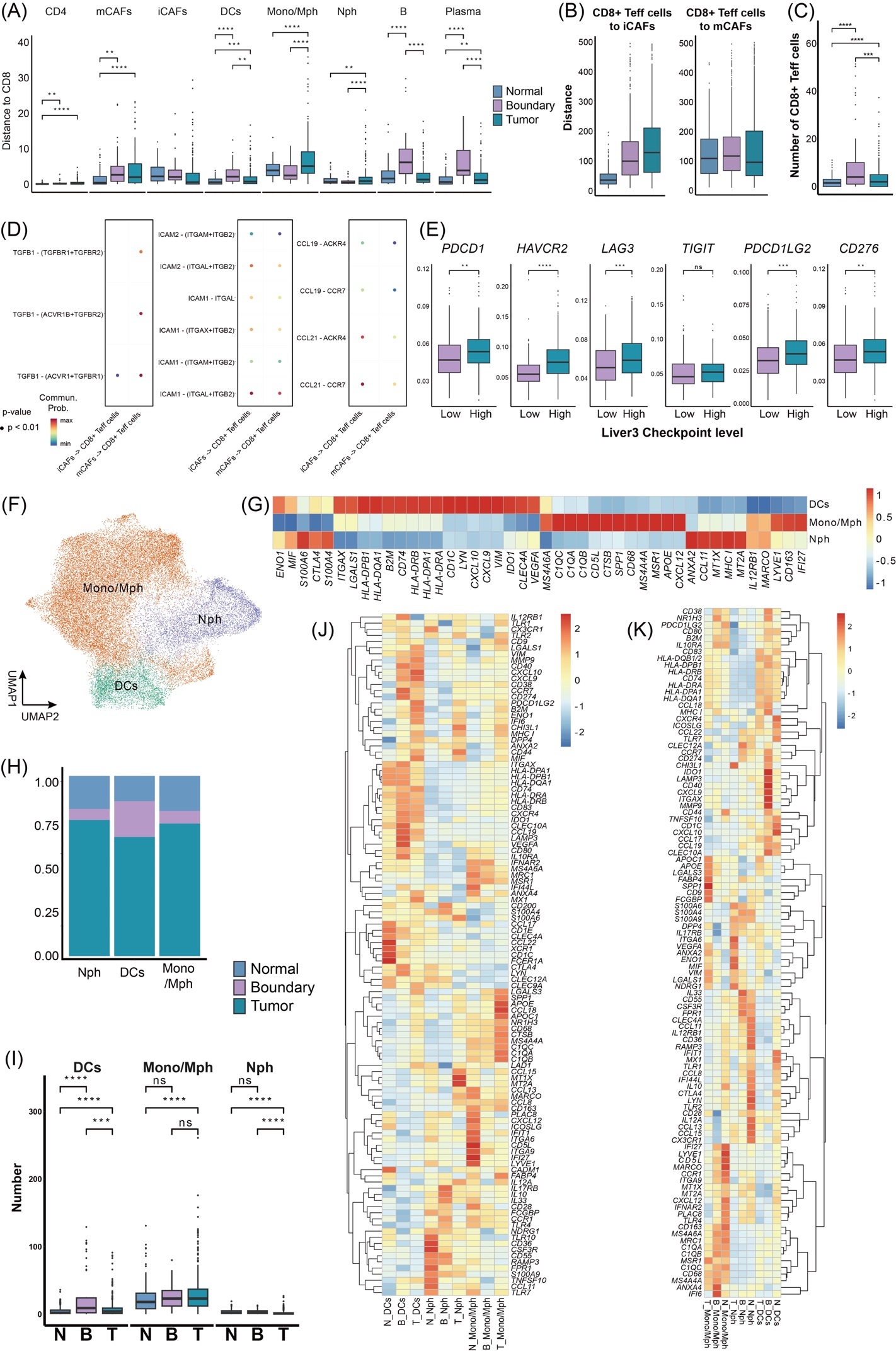


**Figure S8 Comprehensive analysis of immune cell distribution and interactions with CAFs in the TME.**

(A) Distance analysis showing the proximity of CD8^+^ T cell to CAF subtypes and other immune cells across normal (N), boundary (B), and tumoral (T) regions in CosMx6000 data. (B) Distance of CD8⁺ T cells to iCAFs and mCAFs across T, B, N. (C) Counts of CD8⁺ T cells across T, B, N. (D) Analyzing the intercellular signals received by CD8^+^ Teff and comparing the differences between mCAFs and iCAFs in CosMx1000 slide 1. (E) Box plots showing the expression levels of immune checkpoint genes (*PDCD1, HAVCR2, TIGIT, CD276, PDCD1LG2*) in mCAF-high and mCAF-low groups of CosMx6000 data. (F) UMAP visualization for myeloid cells in CosMx6000. (G) Heatmap for myeloid cell types of CosMx6000 data. (H) The stacked barplot of tissue region preference of different myeloid cell subtypes in CosMx1000 data. (I) The boxplot of the absolute cell abundance of different myeloid cell subtypes across Normal (N), Boundary (B), and Tumoral (T) regions in CosMx1000 data. (Supplementary figure for Figure 6I) (J) Heatmap for myeloid cell DEGs in different tissue regions of CosMx6000 data. (K) Heatmap for myeloid cell DEGs in different tissue regions of CosMx1000 data.


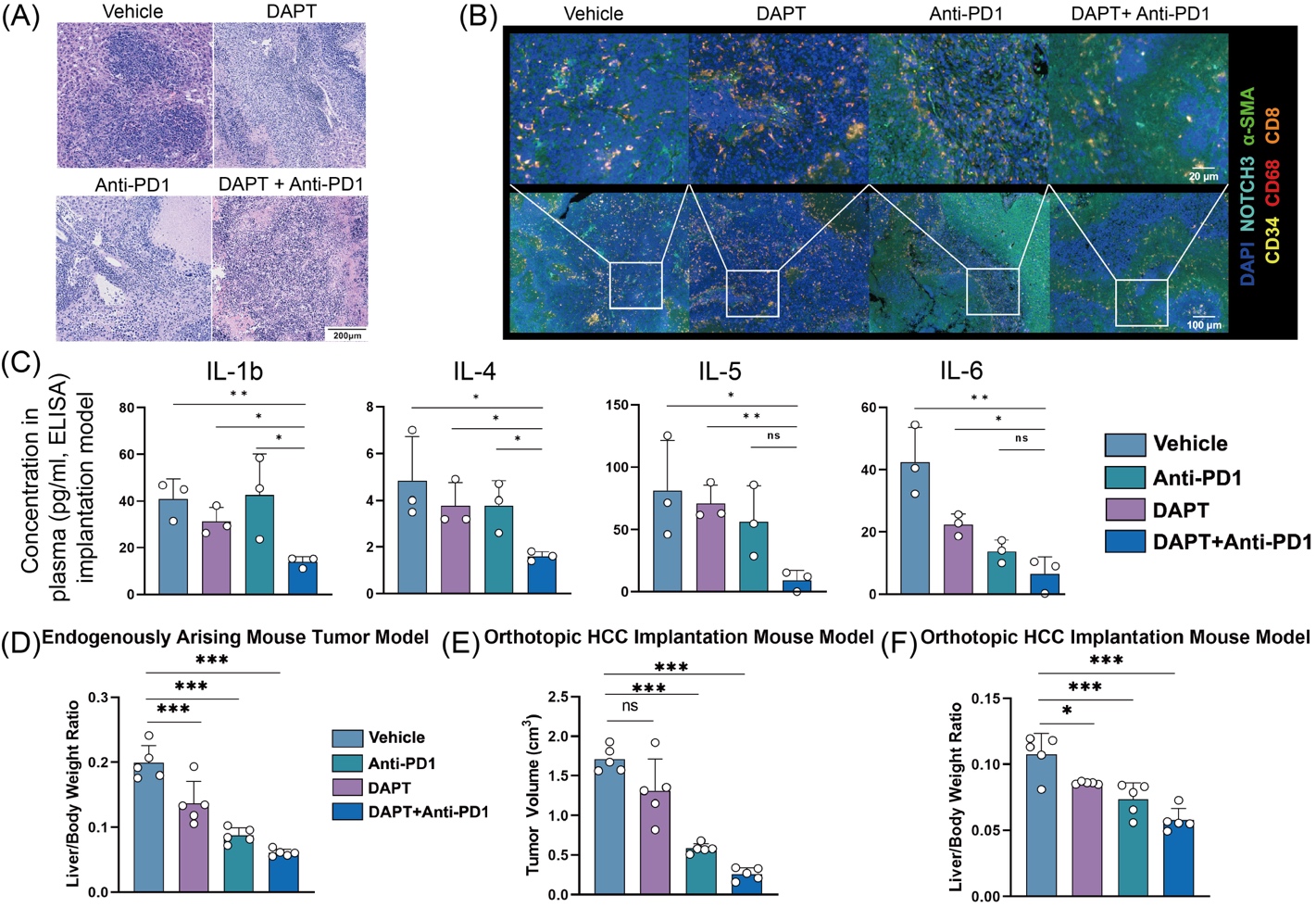


**Figure S9 Additional results of NOTCH-targeted treatment in mouse models.**

(A, B) HE and multiplex immunohistochemical staining images of different treatment groups in the implantation mouse model. (C) Concentration of IL-1b, IL-4, IL-5, and IL-6 in plasma of the orthotopic implantation model. (D) Tumor Volume of orthotopic HCC implantation mouse model between 4 groups. (E) Liver/Body weight ratio of orthotopic HCC implantation mouse model between 4 groups. (F) Liver/Body weight ratio of endogenously arising mouse model between 4 groups.

**Reference**

1. Khemlina, Galina, Sadakatsu Ikeda, Razelle Kurzrock. 2017. “The biology of Hepatocellular carcinoma: implications for genomic and immune therapies.” *Molecular cancer* 16: 149. <https://doi.org/10.1186/s12943-017-0712-x>

2. Ruf, Benjamin, Matthias Bruhns, Sepideh Babaei, Noemi Kedei, Lichun Ma, Mahler Revsine, Mohamed-Reda Benmebarek, et al. 2023. “Tumor-associated macrophages trigger MAIT cell dysfunction at the HCC invasive margin.” *Cell* 186: 3686-3705.e3632. <https://doi.org/10.1016/j.cell.2023.07.026>

3. Stringer, Carsen, Tim Wang, Michalis Michaelos, Marius Pachitariu. 2020. “Cellpose: a generalist algorithm for cellular segmentation.” *Nature Methods* 18: 100–106. <https://doi.org/10.1038/s41592-020-01018-x>

4. Hao, Yuhan, Tim Stuart, Madeline H. Kowalski, Saket Choudhary, Paul Hoffman, Austin Hartman, Avi Srivastava, et al. 2023. “Dictionary learning for integrative, multimodal and scalable single-cell analysis.” *Nature Biotechnology* 42: 293–304. <https://doi.org/10.1038/s41587-023-01767-y>

5. Yeh, Christine Yiwen, Karmen Aguirre, Olivia Laveroni, Subin Kim, Aihui Wang, Brooke Liang, Xiaoming Zhang, et al. 2024. “Mapping spatial organization and genetic cell-state regulators to target immune evasion in ovarian cancer.” *Nature Immunology* 25: 1943-1958. <https://doi.org/10.1038/s41590-024-01943-5>

6. Ji, Fansen, Weitong Bi, Jiawei Zhang, Bingjun Tang, Ying Xiao, Huan Li, Hao Liu, et al. 2025. “scICB: A pan‐cancer database of human temporal immune checkpoint blockade therapy at single‐cell transcriptomic resolution.” *Clinical and Translational Discovery* 5: e70044. <https://doi.org/10.1002/ctd2.70044>

7. Tang, Zefang, Boxi Kang, Chenwei Li, Tianxiang Chen, Zemin Zhang. 2019. “GEPIA2: an enhanced web server for large-scale expression profiling and interactive analysis.” *Nucleic Acids Research* 47: W556-W560. <https://doi.org/10.1093/nar/gkz430>

8. Goldman, Mary J., Brian Craft, Mim Hastie, Kristupas Repečka, Fran McDade, Akhil Kamath, Ayan Banerjee, et al. 2020. “Visualizing and interpreting cancer genomics data via the Xena platform.” *Nature Biotechnology* 38: 675-678. <https://doi.org/10.1038/s41587-020-0546-8>
